# Dentition of the Mugharet El'Aliya Fossil Human Maxilla, Morocco

**DOI:** 10.1002/ajpa.70015

**Published:** 2025-02-22

**Authors:** Carolin Röding, Sireen El‐Zaatari, Fernando V. Ramirez Rozzi, Chris Stringer, M. Loring Burgess, Rodrigo S. Lacruz, Katerina Harvati

**Affiliations:** ^1^ Paleoanthropology, Senckenberg Centre for Human Evolution and Palaeoenvironment, Eberhard Karls University of Tübingen Tübingen Germany; ^2^ Institute for Archaeological Sciences, Eberhard Karls University of Tübingen Tübingen Germany; ^3^ Eco‐Anthropologie (EA), Muséum National d'Histoire Naturelle, CNRS, Université de Paris Paris France; ^4^ Centre for Human Evolution Research, the Natural History Museum London UK; ^5^ Peabody Museum of Archaeology and Ethnology, Harvard University Cambridge Massachusetts USA; ^6^ Department of Molecular Pathobiology New York University College of Dentistry New York New York USA; ^7^ DFG Centre of Advanced Studies ‘Words, Bones, Genes, Tools’, Eberhard Karls University of Tübingen Tübingen Germany

**Keywords:** Aterian, enamel‐dentine junction, human evolution, Middle Stone Age, Northwest Africa

## Abstract

**Objective:**

This study follows up on our recent morphological analysis of the juvenile maxilla from Mugharet el'Aliya, Morocco. Although this specimen shows a reportedly archaic morphology, likely due to its large size, 3D shape analyses indicated affinities with early *Homo sapiens* . Here, we conducted an in‐depth comparative investigation of the associated dentition to further clarify this individual's phylogenetic and taxonomic affinities.

**Materials and Methods:**

Our analyses were based on three kinds of data: (a) external crown dimensions and non‐metric features, analyzed via summary statistics; (b) CT scan data enabling the study of internal structures (enamel‐dentine junction) via geometric morphometrics; and (c) high‐resolution replicas of the external surface of the upper canine enabling the study of perikymata numbers via probability functions. The comparative samples included Middle Pleistocene (Chibanian) Europeans and Africans, Neanderthals, and early and later *H. sapiens* .

**Results:**

Mugharet el'Aliya showed the greatest similarities in external and internal tooth morphology with early and later *H. sapiens.* Perikymata counts cluster the upper canine with *H. sapiens* . However, its canine and fourth premolar are megadont at a level generally atypical for *H. sapiens.*

**Discussion:**

Our analyses of the dentition of the Mugharet el'Aliya individual support our previous findings on the morphological analysis of the maxilla, placing this fossil closest to *H. sapiens* . Our study further strengthens the evidence connecting fossils from the North African Aterian to those from Western Asia, especially Qafzeh. We also provide the first comparative analysis of a permanent upper canine from the Aterian fossil record.

## 1 Introduction

Morocco boasts a rich Middle and Late Pleistocene hominin fossil record, often associated with the Middle Stone Age (MSA; e.g., Hublin et al. 2012; Smith et al. 2007; Scerri 2017). Many of these fossils show a mixture of primitive and derived traits, with robust jaws and high levels of megadontia particularly in the post‐canine dentition, making it difficult to infer their taxonomic classification (e.g., Ferembach 1976; Hublin and Tillier 1981; Smith et al. 2007; Hublin et al. 2012). Additionally, historical views had a residual effect on the interpretations of the results of their analyses, and thus on their integration into an overarching framework. During the first half of the 20th century, fossil human remains associated with Northwest African MSA assemblages were often attributed to Neanderthals (e.g., Şenyürek 1940; McBurney et al. 1953; Ennouchi 1962), an interpretation that reflected prevalent views of both the North African and European human fossil record at the time (for a recent discussion see Harvati and Reyes‐Centeno 2022). Similarly, the Aterian lithic industry was considered to be associated with a Neanderthal‐like population producing late MSA lithic assemblages between 40 and 20 ka (see discussion in e.g., Dibble et al. 2013; Campmas 2017; Scerri 2017).

Today, the fossils from Jebel Irhoud, Morocco, dating to ca. 300 ka, are widely recognized as the earliest known members of the *Homo sapiens* lineage (Richter et al. 2017; Hublin et al. 2017). Several other geologically younger Moroccan fossils associated with the Aterian lithic industry have also been found to likely represent early *H. sapiens* as they show morphological similarities not only to the Jebel Irhoud specimens but also to Levantine samples from the sites of Skhul and Qafzeh (e.g., Hublin and Tillier 1988; Harvati and Hublin 2012; Hublin et al. 2012; Freidline et al. 2024; Röding et al. 2023). Furthermore, the interpretations of the Aterian techno‐complex have also shifted over recent decades. Nowadays, it is described as an MSA industry with features like personal ornaments and pigments, most likely produced by early *H. sapiens* between ca. 145–20 ka (e.g., Barton et al. 2009; d'Errico et al. 2009; Richter et al. 2010; Hublin et al. 2012; Doerschner et al. 2016). In this context, investigating the taxonomy and phylogenetic position of fossil hominins associated with the Aterian is critical for better understanding the evolutionary processes underlying modern human origins in the region.

We aim to contribute to this investigation through an in‐depth comparative assessment of the dentition of the juvenile fossil from Mugharet el'Aliya, Morocco. Our recent analysis of the maxillary morphology of this specimen indicated affinities with early *H. sapiens* , suggesting that its previously perceived archaic morphology is mainly a by‐product of its large size (Röding et al. 2023). Here we build on that analysis with the investigation of the dental remains from the same individual. Dental morphometric features, both external and internal, have been shown to be highly informative on phylogeny and taxonomy (e.g., Bailey 2002, 2006; Rathmann et al. 2017, 2023), and can be used to help elucidate the affinities of fossil specimens (e.g., Benazzi et al. 2011; Harvati et al. 2003, 2013, 2015; Röding et al. 2021; Zanolli et al. 2022; Skinner et al. 2008). Further, dental growth patterns in the form of perikymata packing patterns (PPP) may be used to provide additional information on taxonomy. Perikymata are the external manifestation of long‐period incremental features in the tooth that reflect periodic growth intervals during enamel formation (e.g., Smith and Tafforeau 2008; Risnes 1998). Perikymata counts, especially their pattern across specific crown segments, so‐called deciles, are known to differentiate between hominin groups (e.g., Dean and Reid 2001; Modesto‐Mata et al. 2020; Ramirez Rozzi and Bermúdez de Castro 2004). We, therefore, extended our analysis of the Mugharet el'Aliya individual to its external and internal dental morphology as well as dental growth patterns to further clarify this individual's phylogenetic and taxonomic affinities. In this context we (1) examine if the dentition shares the maxilla's *H. sapiens* ‐like morphology coupled with an exceptional size, and (2) investigate this individual's similarities to additional Aterian dental material from Morocco.

### 1.1 Mugharet El'Aliya: Background

Mugharet el'Aliya (hereafter el'Aliya) is a cave site on the Moroccan Atlantic coast (35°45′ N, 5°56′ W; Wrinn and Rink 2003) near Tangier, with deposits spanning from the later MSA to the Roman‐Islamic periods (Bouzouggar et al. 2002). The later MSA layers and materials are ascribed to the Aterian period and were first excavated in 1939 (Coon 1957a; Bouzouggar et al. 2002). At the same time, two hominin remains were also recovered: a left maxillary fragment of a juvenile with three unerupted teeth and an isolated molar (Coon in Şenyürek 1940; Coon 1957a; Şenyürek 1940). The maxillary fragment was presumably recovered during the sieving of unmixed sand from the lowest and oldest layer, which also yielded the Aterian tools (Coon in Şenyürek 1940; Coon 1957a; Howe 1967; Bouzouggar et al. 2002; Wrinn and Rink 2003), but this remains uncertain (Coon 1957b; Minugh‐Purvis 1993). Today, the cave is almost completely emptied of its sediments, preventing a clear understanding of the exact provenance and geological age of the fossil (Bouzouggar et al. 2002; Wrinn and Rink 2003). Indirect dating of faunal remains provides a broad chronological context for the maxilla and associated teeth to between 35 and 60 ka (Wrinn and Rink 2003; for a more detailed discussion see Röding et al. 2023).

The reportedly archaic morphology of the maxilla led to its previous attribution to the Neanderthal lineage (Şenyürek 1940). However, more recent studies have suggested affinities with early *H. sapiens* , while highlighting that its perceived archaic morphology is mainly related to its large size (Minugh‐Purvis 1993; Röding et al. 2023).

### 1.2 The Dentition

**FIGURE 1:**
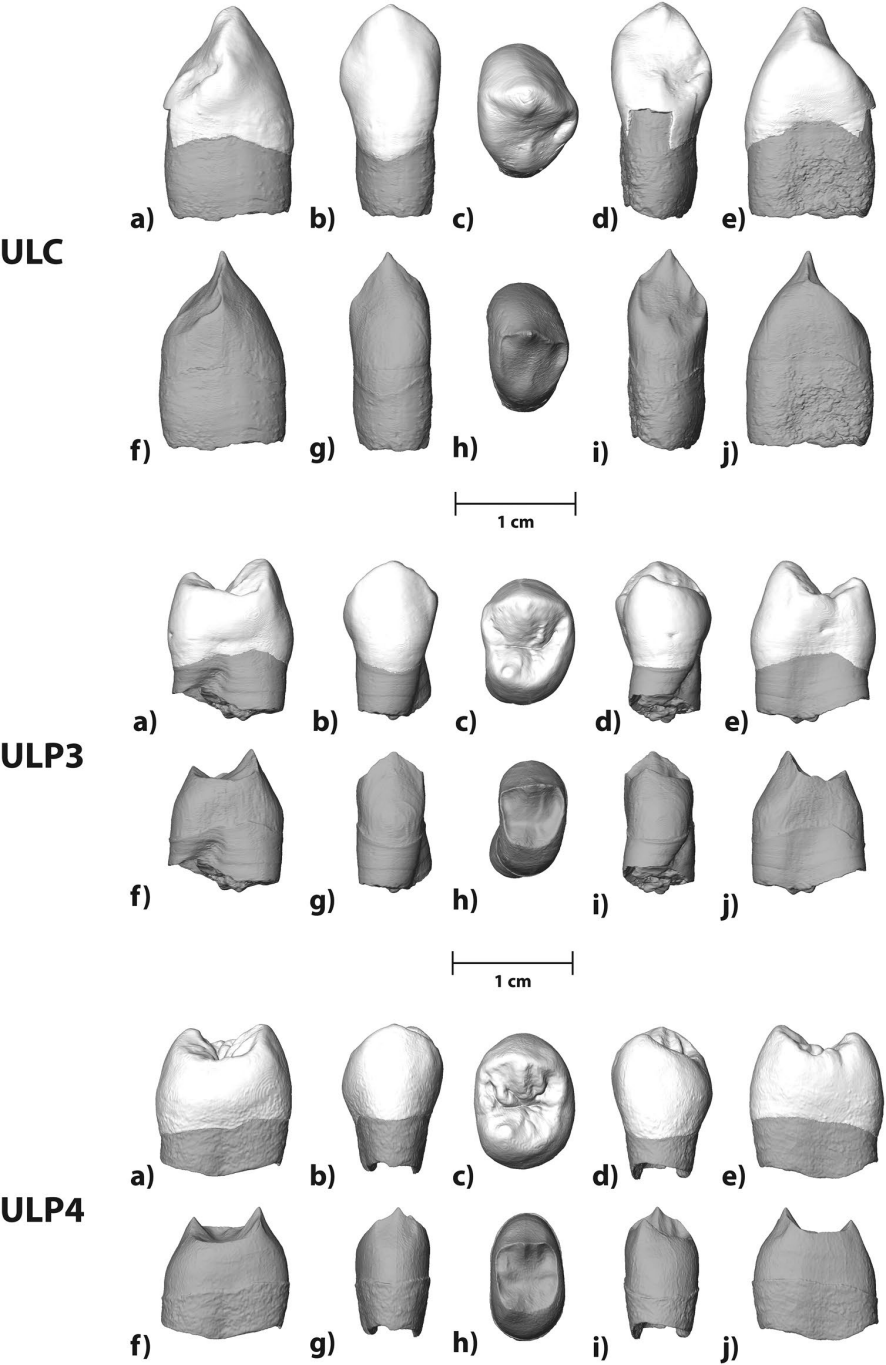
Three‐dimensional models of the ULC, ULP3, and ULP4 from Mugharet el'Aliya. In the respective upper row, each tooth crown is shown in (a) distal, (b) buccal, (c) occlusal, (d) lingual, and (e) mesial view. In the respective bottom row, each dentine surface is shown in (f) distal, (g) buccal, (h) occlusal, (i) lingual, and (j) mesial view. Occlusal views are shown with buccal to the top; lingual to the bottom, distal to the right, and mesial to the left. Mugharet el'Aliya is courtesy of the Peabody Museum of Archaeology and Ethnology, Harvard University, 39‐69‐50/N3635.0.

Here we analyzed the three teeth belonging to the el'Aliya maxilla: an upper left permanent canine (ULC), a third premolar (ULP3), and a fourth premolar (ULP4; Figure 1). All teeth were unerupted at the time of death; their crowns are completely formed, but their roots were still in the process of formation. Based on recent *H. sapiens* standards for dental development, the age at death of this individual is estimated at 8–9 years (AlQahtani et al. 2010; cf. Şenyürek 1940; Minugh‐Purvis 1993).

During the initial description of the specimen, the ULC and ULP3 were removed from their crypts, and detailed descriptions were provided (Şenyürek 1940). In contrast, the ULP4 remains in its crypt and has never been described. Here, we provide the first description of this tooth, relying on a high‐resolution computer tomography (CT) scan. We also provide updated descriptions for the ULC and ULP3, relying on high‐resolution CT scans, as well as comparative analyses of all three teeth, including enamel‐dentine junction shape, crown morphology, and perikymata packing pattern.

## 2 Materials and Methods

### 2.1 Datasets and Sample Composition

**FIGURE 2:**
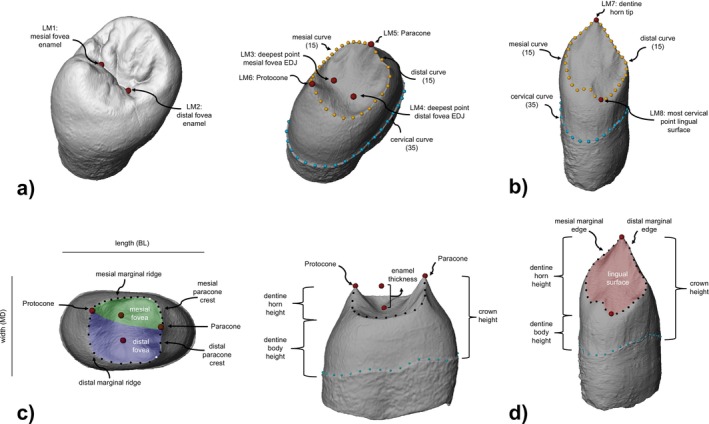
Landmarking protocol and terminology guide. Example of the landmarking protocol for (a) the premolars, shown by the example of a UP4, and (b) a UC. LM Numbers refer to landmark definitions provided in SOM Table S5 and mark fixed landmarks. Numbers in brackets indicate the number of equidistant semi‐landmarks along each curve. Illustration of main features and the here used corresponding terminology shown in (c) for the premolars and (d) a UC. Figure not to scale. Mugharet el'Aliya is courtesy of the Peabody Museum of Archaeology and Ethnology, Harvard University, 39‐69‐50/N3635.0.

The el'Aliya subadult maxilla and associated teeth (39‐69‐50/N3635.0) are housed at the Peabody Museum of Archaeology and Ethnology, Harvard University, Cambridge, USA. Here we focus on the dentition, for which we collected three kinds of data: (a) external crown dimensions and non‐metric features, (b) internal morphological data from the enamel‐dentine junction (hereafter EDJ), and (c) perikymata counts. The first two datasets were retrieved for all three teeth from CT scans, whereas the latter was retrieved only for the UC from positive high‐resolution dental casts of the original teeth. Key terminology for all analyses and subsequent discussion of their results is illustrated in Figure 2.

The el'Aliya dental morphology was then assessed in a comparative framework. Our comparative samples comprised Middle Pleistocene individuals (~MIS 16‐7) from Northwest Africa (Morocco and Algeria), Middle Pleistocene pre‐Neanderthals (~MIS 12‐7) from Europe, *H. neanderthalensis* (MIS 7‐3), and *H. sapiens* . The latter were further subdivided into early *H. sapiens* (> MIS 4 to MIS 9), Aterian, and later *H. sapiens* (< MIS4; see Table 1 for details on comparative sample compositions for analyses of each dataset). For the UC perikymata analysis, the *H. sapiens* and Middle Pleistocene Europe samples exclusively comprise European Upper Paleolithic individuals and individuals from Sima de los Huesos, respectively.

**TABLE 1:** Sample overview for all performed analyses. Groups marked with x or labeled pooled were included in the respective analyses. The table includes references to supplementary information (SOM Tables S2, S3, S4, and S6), which provide further information on sample compositions and sources for data from the literature for each respective analysis.

Group		Additional information	Analyses							
			External non‐metric features		External crown dimensions		Enamel‐Dentine junction (EDJ)		Perikymata packing pattern (PPP) UC	
			Groups	Fossil sample (abbreviation)	Groups	Fossil sample	Groups	Fossil sample (abbreviation)	Groups	Fossil sample
Mugharet el'Aliya			x	El'Aliya	x	El'Aliya	x	El'Aliya	x	El'Aliya
*Homo sapiens*	Aterian		x	Grotte des Contrebandiers T5 (T5), Dar‐es‐Soltane II—H6 (DS6)	x	Data from literature		Grotte des Contrebandiers T5 (T5), Dar‐es‐Soltane II—H6 (DS6)		
	Early	> MIS 4 to MIS 9	x	Data from literature	x	Data from literature	Pooled	Jebel Irhoud 21 (IR21), Qafzeh 10 (QA10), Qafzeh 15 (QA 15)		
	Later	European Upper Paleolithic individuals	Pooled	Data from literature	Pooled	Data from literature	Pooled	Abri Pataud (AP), La Rochette (LR)	x	Abri Pataud 22, Aurensan di, Grotte de Bedeihac, Estagel 1, Laugerie‐Basse, Le Placard, Mas d'Azil, Saulges Mx 1, Solutré (Mx 2, di), St. Germain‐la‐Rievière 10, Tarté 1
		Epi‐Paleolithic North Africa, Mesolithic, Neolithic								
*Homo neanderthalensis*		MIS 7‐3 includes Middle Pleistocene individuals from Krapina	x	Data from literature	x	Data from literature	x	Abri Bourgeois‐Delaunay 14 (BD14), Krapina (Kr: 39, 41, 42, 44, 45, 46, 47, 49, 53, 76, 102, 103, 112, 115, 116, 117, 141, 191), La Quina H18 (LQ18), Le Moustier (LM)	x	Genay 1, Hortus (II‐III, VIII, IX), Krapina (36, 37, 56, 76, 103, 139, 141, 142, 144, 146, 147, Mx 45.1, Mx E, Mx F), La Ferrassie II, La Quina (5, 17), Monsempron II, Saccopastore II, Vindija 12.5
Middle Pleistocene Europe		Pre‐Neanderthals ~MIS 12‐7	x	Data from literature	x	Data from literature	x		x	Sima de los Huesos: 44, 94, 144, 163, 558, 818, 825, 955, 958, 1475, 1757, 1758, 1942, 2151, 2207, 2388. 2392, 3191
Middle Pleistocene NW Africa		~MIS 16‐7 from Morocco and Algeria			x	Data from literature	x			
Further details and sources			SOM Table S2		SOM Table S3		SOM Table S4		SOM Table S6	

### 2.2 CT Scans Mugharet El'Aliya

CT scans of the el'Aliya dentition were acquired with the HMXST Micro‐CT X‐ray imaging system at the Center for Nanoscale Systems (CNS) at Harvard University. The UC and UP3 were scanned without a filter, at 85 kV and 125 μA with an isotropic voxel size of 9.696 and 9.311 μm, respectively. The maxilla and, thereby, the UP4 were scanned with a 0.5 mm copper filter, at 105 kV and 180 μA with an isotropic voxel size of 26.242 μm.

### 2.3 External Crown Morphology

The external crown measurements and features for all three el'Aliya teeth were acquired from 3D surfaces of the entire tooth crowns extracted from the CT scans. Crown morphologies for each of the three teeth were described and scored using a set of non‐metric traits. Definitions, grades, and descriptions of individual non‐metric traits are listed in SOM Table S1 and are based on Martinón‐Torres et al. (2012), Bailey (2006), and Scott and Irish (2017). The scored non‐metric traits were then compared to previously published data (Table 1; SOM Table S2).

Buccolingual (BL) and mesiodistal (MD) crown dimensions of the el'Aliya teeth were virtually measured (in mm) and compared to data from the literature, which was supplemented by raw measurements collected and kindly provided by S.E.B. (Table 1; SOM Table S3).

For both datasets, summary statistics were calculated. These include mean ($\bar{x}$) and standard deviation (*σ*) for crown dimensions and percentages for non‐metric traits.

### 2.4 Enamel–Dentine Junction (EDJ) Datasets

Only individuals with fully developed tooth crowns and showing wear facets in stage 1 (unaffected dentine) or stage 2 (with a minimal amount of dentine missing at the cusps), according to Smith (1984), were included in the EDJ samples. A total of 93 CT scans were used in three EDJ datasets: UC, UP3, and UP4 (Table 1; SOM Table S4).

#### 2.4.1 Measurement Protocol

In the first step, we virtually segmented each tooth, both from el'Aliya and the comparative samples, into enamel and dentine via semi‐automated segmentation along the EDJ. This step included threshold‐based segmentation in all slices (Avizo::Magic Wand) followed by manual corrections (Avizo::Brush) in areas of the CT scan with similar density and, thereby, similar gray values between enamel and dentine. Missing dentine horn tips were reconstructed based on the curvature of the remaining dentine by extrapolating the curvature to reconstruct their intersection at the tip (cf. e.g., Smith et al. 2012; Pan et al. 2020). For data collection, we created three‐dimensional surface models (hereafter: surfaces) of both the entire tooth crown and the dentine along the EDJ. Extracted surfaces from the right teeth were mirrored to be comparable to left teeth in order to increase the sample size.

A set of six fixed landmarks (dentine: paracone, protocone, and the deepest points of mesial and distal fovea; enamel: mesial and distal fovea) and 65 semi‐landmarks representing three curves (dentine: mesial and distal marginal ridges, and the cervical line) were digitized on each premolar (Figure 2a; SOM Table S5). Placement of the landmarks took place on the superimposed extracted surfaces of the entire crown and dentine, with landmarks being placed directly on the crown and dentine surfaces, respectively. By covering the EDJ as well as the occlusal surface of the enamel, the landmark set includes indirect measures of enamel thickness at the mesial and distal foveae (cf. Figure 2c). For the inclusion of landmarks along the enamel, the fovea landmarks were chosen due to the combination of their inclusion in previous studies (e.g., Gómez‐Robles et al. 2011; Xing et al. 2021) and the fact that dental wear needs to exceed stage 2, according to Smith (1984), to affect these landmarks.

The dataset for the UC included two fixed landmarks (dentine: horn tip and the most cervical point on lingual surface) and 65 semi‐landmarks representing three curves (dentine: mesial and distal edges of the lingual surface, and the cervical line) (Figure 2b; SOM Table S5) and was collected entirely on the extracted dentine surface. The fixed landmark at the lingual surface is here defined as the most cervical point along the intersection of curves along the mesial and distal edges of the lingual surface. In the case of distinct mesial and/or distal ridges, these curves also capture the ridge morphology in addition to outlining the lingual surface. Further, in the presence of a marked tuberculum dentale, the cervical point was collected distally to the tubercle because the intersection between the tubercle and the distal edge of the lingual surface tends to be more cervically located than the mesial counterpart in our sample. Thereby, the mesial curve captures information regarding the manifestation of the tuberculum dentale.

The three curves of all datasets were digitized as densely spaced points on the dentine surfaces and resampled to their respective number of predefined landmarks. The cervical curve along the cementum–enamel junction was treated as a closed curve beginning on the buccal side below the dentine horn and moving in a mesial direction. The distance between the cervical curve and all other landmarks placed on the EDJ provides an indirect measure of crown height included in the landmark sets (cf. Figure 2c,d).

Semi‐landmarks were allowed to slide along tangent vectors to the curves and tangent planes to the surfaces by minimizing the bending energy, following established protocol (Gunz et al. 2005). The raw landmark configurations were superimposed with generalized Procrustes analysis (GPA), which removes information about orientation and location and scales them to centroid size (CS; square root of the summed squared of each landmark‐centroid distance) (Zelditch et al. 2012), allowing for the statistical analysis of the resulting Procrustes shape coordinates (see e.g., Gunz et al. 2005; Harvati et al. 2019; Zelditch et al. 2012).

#### 2.4.2 Error Calculations

Multiple measurements of the same individual were carried out to obtain intra‐ and interobserver error estimates. Two observers collected five repeated measurements each (a total of 10) for each landmark set (UC, UP3, and UP4) over a period of three months (cf. Figure 2a,b; SOM Table S5). One observer can be characterized as experienced (C.R.) and one as naïve (J.Z.), with the latter not regularly working on dental samples. Error was calculated (1) based on Procrustes distances (PD) of the entire datasets including curve semi‐landmarks between all individuals (e.g., Neubauer et al. 2010), and (2) as Euclidean distances (ED) between the repeated measurements of each fixed landmark position (e.g., Percival et al. 2019). The latter was calculated in mm at each landmark position rather than as the distance from each landmark to the centroid, as this can be problematic in non‐spherical landmark configurations (for discussion see, e.g., von Cramon‐Taubadel et al. 2007).

The largest pairwise PD between repeated measurements was smaller than the smallest PD between different individuals. Further, the Euclidean distances between repeated measurements do not exceed 0.5 mm at each landmark position, including the ones on the enamel of the premolars (SOM Figures S1 and S2). The average intra‐ and interobserver error at each landmark position falls between 0.05 and 0.16 mm. To visualize the effect of this error, principal component analyses (PCA) were calculated. The PCAs were calculated based on the comparative sample, and all repeated measurements were projected into the plots (see also, e.g., Harvati et al. 2019; Harvati et al. 2024; Röding et al. 2021). The plots show that all repeated measurements cluster closely together and no other individual overlaps with their variation (SOM Figure S3). Therefore, we do not expect the intra‐ and inter‐observer errors to significantly influence our results (cf. e.g., Percival et al. 2019; Neubauer et al. 2010).

#### 2.4.3 Analyses

We performed PCAs of the Procrustes shape variables, with the el'Aliya, early *H. sapiens* and Aterian specimens projected into the plots (see also, e.g., Harvati et al. 2019; Harvati et al. 2024; Röding et al. 2021). Calculating PCAs based on the entire comparative sample and only projecting el'Aliya affects the position of individuals but does not alter the overall pattern (cf. SOM Figure S3; Results section). We visualized shape changes along each plotted principal component (PC) as surfaces based on the deformations of the landmark set by ±2 standard deviations (sd). To statistically assess allometry, we conducted linear regressions of shape and log CS. Statistical significance was assessed through 10,000 permutations. We also plotted log CS against shape in the form of PCs to visualize their allometric relationship. All statistical tests were considered significant at *α* ≤ 0.05.

### 2.5 Perikymata Analysis (UC Only)

We analyzed the perikymata packing pattern (PPP) of the el'Aliya UC following the protocol described in Ramirez Rozzi and Bermúdez de Castro (2004). The ULC was molded using Coltène President light putty (Coltène/Whaledent AG, Feldwiesenstrasse, Altstätten, Schweiz), and a positive cast was made using Araldite 2020 (Huntsman Corporation, The Woodlands, TX, USA). The buccal face of the cast was oriented perpendicular to the light microscope axis, and the crown height, measured from the cervix to the apex, was divided into 10 deciles. The number of perikymata was obtained in those deciles where perikymata were clearly observable. These counts were compared with data from equivalent deciles in UCs from published samples (Table 1; SOM Table S6; cf. Ramirez Rozzi and Bermúdez de Castro 2004). The obtained perikymata counts were used to calculate summary statistics including mean $\left(\bar{x}\right)$, standard deviation (*σ*), and range. Furthermore, probability functions and resulting plots were generated.

### 2.6 Software

Avizo (version 9.2 Lite; Visualization Science Group) was used to segment and extract surfaces, as well as to obtain fixed landmarks and curve semi‐landmarks as part of the EDJ measurement protocol. All other steps of the EDJ measurement protocol, the analyses of the EDJ datasets, dental dimensions, and non‐metric traits were carried out in R (R Developmental Core Team 2022) by mainly using the R packages geomorph (version 4.0.5; Adams et al. 2022), Morpho (version 2.11; Schlager 2017), Arothron (version 2.0.5; Profico et al. 2023), and MASS (version 7.3–58.3; Venables and Ripley 2002). SYSTAT (version 10.2; Systat Software Inc.) was used to generate the probability functions and plots based on the perikymata dataset. All other graphics were created in R and processed in Adobe Illustrator CS5.

## 3 Results

### 3.1 External Crown Morphology

#### 3.1.1 ULC

When viewed lingually, the canine crown shows mild shoveling (grade 1, cf. SOM Tables S1 and 2). The mesial and distal marginal ridges are slightly thickened and very similar in size (grade 1), with the mesial one being slightly larger. They do not connect with the weak *tuberculum dentale* (grade 1). A weakly developed distal accessory ridge is present (grade 1).

**TABLE 2:** Frequencies of the degrees of expression of upper permanent canine (UC) main morphological traits. Description of traits in SOM Table S1. Bold values indicate traits for which 100% of the studied individuals in a group exhibit the same grade.

Traits	Grade	El'Aliya	Middle Pleistocene Europe	*Homo neanderthalensis*	*Homo sapiens*	
					Early	Later
Tuberculum dentale	0		1 (4.2%)	0	5 (83.3%)	93 (73.9%)
	1	x	3 (12.5%)	0		
	2		4 (16.6%)	0	1 (16.7%)	28 (23.1%)
	3		7 (29.2%)	6 (28.6%)		
	4		2 (8.4%)	5 (23.8%)		
	5		3 (12.5%)	7 (33.3%)		
	6		4 (16.6%)	3 (14.3%)		
	*Total*		24	21	6	121
Shovel shape	0		0	0	1 (16.7%)	71 (59.2%)
	1	x	0	0		
	2		0	0	5 (83.3%)	49 (40.8%)
	3		5 (20%)	9 (40.9%)		
	4		13 (52%)	9 (40.9%)		
	5		7 (28%)	4 (18.2%)		
	*Total*		25	22	6	120
Canine mesial ridge	0		2 (9.5%)	5 (26.3%)	**6 (100%)**	109 (94%)
	1	x	4 (19%)	6 (31.6%)		
	2				0	7 (6%)
	3		15 (71.5%)	8 (42.1%)		
	*Total*		21	19	6	116
Distal accessory ridge	0		8 (50%)	9 (56.3%)	1 (25%)	61 (64.9%)
	1	x	3 (18.7%)	4 (25%)	3 (75%)	33 (35.1%)
	2					
	3					
	4		5 (31.3%)	3 (18.8%)		
	5					
	*Total*		16	16	4	94
Source			a	a	b,c,d	a,b

*Note:* (a) Martinón‐Torres et al. (2012, table 9); (b) Bailey (2006, table 3); (c) Hublin et al. (2017), Jebel Irhoud; (d) Hershkovitz et al. (2018), Misliya.

#### 3.1.2 ULP3

This tooth has an oval outline that is mesiodistally compressed, and the buccal part is broader than the lingual one. The buccal cusp (paracone) is bigger and slightly taller than the lingual one (protocone). The apex of the paracone falls on the crown midline, whereas that of the protocone is very much mesially deviated in relation to the paracone. The occlusal surface has few ridges and fissures. The distal and mesial marginal ridges are present. A mesial marginal developmental groove is present; it crosses the mesial marginal ridge and ends on the mesial surface of the crown. A small tubercle on the mesial side is present (grade 1, cf. SOM Tables S1 and 3). The two essential (lingual and buccal) crests are present and unbifurcated (both grade 1): the buccal one is faint, whereas the lingual one is more marked. No transverse crest is present (grade 0). Two unbifurcated distal accessory ridges (MaxPAR) are identified: a weak one on the protocone (not scored here, cf. SOM Table S1) and a very faint one on the paracone (grade 1). On the mesial side, no accessory ridges are present (grade 0). A well‐defined central groove extends from a mesial fovea to a smaller, more buccally located distal fovea. Only one very faint accessory groove on the distolingual side is present.

**TABLE 3:** Frequencies of the degrees of expression of the upper permanent third premolar (UP3) main morphological traits. Description of traits in SOM Table S1. Bold values indicate traits for which 100% of the studied individuals in a group exhibit the same grade.

Traits	Grade	El'Aliya	Middle Pleistocene Europe	*Homo neanderthalensis*	*Homo sapiens*		
					Aterian	Early	Later
Buccal essential crest	0		8 (42.1%)	1 (6.7%)	0	0	1 (0.8%)
	1	x	6 (31.6%)	6 (40.0%)	**1 (100%)**	2 (66.7%)	112 (87.5%)
	2		5 (26.3%)	8 (53.3%)	0	1 (33.3%)	15 (11.7%)
	*Total*		19	15	1	3	128
Lingual essential crest	0		0	0	0	0	1 (0.8%)
	1	x	17 (81.0%)	5 (33.3%)	**1 (100%)**	2 (66.7%)	121 (95.3%)
	2		4 (19.0%)	10 (66.7%)	0	1 (33.3%)	5 (3.9%)
	*Total*		21	15	1	3	127
Transverse crest	0	x	16 (72.7%)	14 (93.3%)	**1 (100%)**	**1 (100%)**	**128 (100%)**
	1		5 (22.7%)	1 (6.7%)	0	0	
	2		1 (4.6%)	0	0	0	
	*Total*		22	15	1	1	128
Distal accessory ridge^a^ (on buccal cusp)	0		11 (68.8%)	7 (63.3%)		**1 (100%)**	94 (89.5%)
	1	x	5 (31.2%)	4 (36.4%)		0	11 (10.5%)
	*Total*		16	11		1	105
Mesial accessory ridge (on buccal cusp)^a^	0	x	15 (93.8%)	10 (90.9%)	**1 (100%)**	**1 (100%)**	92 (86%)
	1		1 (6.2%)	1 (9.1%)	0	0	15 (14%)
	*Total*		16	11	1	1	107
Distal accessory cusp	0	x	**1 (100%)**	7 (36.8%)		**4 (100%)**	100 (80%)
	1		0	12 (63.2%)		0	25 (20%)
	*Total*		1	19		4	125
Mesial accessory cusp	0		1 (50%)	12 (63.2%)	**1 (100%)**	3 (75%)	84 (65.6%)
	1	x	1 (50%)	7 (36.8%)	0	1 (25%)	44 (34.4%)
	*Total*		2	19	1	4	128
Source			a,b	a,b	c^b^	b,d	a,b

*Note:* (a) Martinón‐Torres et al. (2012, table 10); (b) Bailey (2002, table 5.1), (c) Hublin et al. (2012), Dar es Soltane II (DS6); (d) Hershkovitz et al. (2018), Misliya.

^a^ In Bailey (2002) distal and mesial accessory ridges were pooled and not reported here.

^b^ Supplemented with our own observations.

#### 3.1.3 ULP4

Overall, this tooth is similar to the ULP3. It has an oval outline and is mesiodistally compressed, with the buccal half being broader than the lingual one. The paracone and protocone are of similar height, with the paracone being slightly higher. The paracone shows a larger cusp area, and its tip is located on the crown midline, while the smaller protocone is placed mesially relative to the paracone and crown midline. Distal and mesial marginal ridges are present, with the mesial ridge being slightly higher relative to the distal ridge. In addition, two unbifurcated essential crests, lingual and buccal, are present (both grade 1, cf. SOM Tables S1 and 4). No transverse crest is present (grade 0) and the cusps are separated by a well‐defined central groove. The central groove intersects with smaller accessory grooves, including mesiobuccal and distobuccal triangular grooves, and ends in the mesial and distal foveae. A mesial marginal developmental groove is present. It crosses the mesial marginal ridge and ends on the mesial surface of the crown. Two MaxPAR are present on the paracone, one distal and one mesial, and several small ridges on the protocone (grade 1, cf. SOM Tables S1 and 4). A small tubercle on the mesial side is present (grade 1).

**TABLE 4:** Frequencies of the degrees of expression of the upper permanent fourth premolar (UP4) main morphological traits. Description of traits in SOM Table S1. Bold values indicate traits for which 100% of the studied individuals in a group exhibit the same grade.

Traits	Grade	El'Aliya	Middle Pleistocene Europe	*Homo neanderthalensis*		*Homo sapiens*	
					Aterian	Early	Later
Buccal essential crest	0		0	0	0	0	1 (0.9%)
	1	x	15 (62.5%)	5 (33.3%)	**1 (100%)**	**3 (100%)**	98 (89.9%)
	2		9 (37.5%)	10 (66.6%)	0	0	10 (9.2%)
	*Total*		24	15	1	3	109
Lingual essential crest	0		0	0	0	0	0
	1	x	11 (45.8%)	3 (21.4%)	**2 (100%)**	2 (66.7%)	99 (91.7%)
	2		13 (54.2%)	11 (78.6%)	0	1 (33.3%)	9 (8.3%)
	*Total*		24	14	2	3	108
Transverse crest	0	x	22 (84.6%)	14 (87.5%)	**2 (100%)**	**1 (100%)**	**113 (100%)**
	1		4 (15.4%)	1 (6.2%)	0	0	0
	2		0	1 (6.2%)	0	0	0
	*Total*		26	16	2	1	113
Distal accessory ridge (on buccal cusp)^a^	0		8 (44.4%)	7 (58.3%)		**1 (100%)**	70 (79.5%)
	1	x	10 (55.6%)	5 (41.7%)	**1 (100%)**	0	18 (20.5%)
	*Total*		18	12	1	1	88
Mesial accessory ridge (on buccal cusp)^a^	0		10 (52.6%)	8 (88.8%)		**1 (100%)**	74 (80.4%)
	1	x	9 (47.4%)	1 (11.2%)		0	18 (19.6%)
	*Total*		19	9		1	92
Distal accessory cusp	0	x	**1 (100%)**	11 (61.1%)	**1 (100%)**	**6 (100%)**	83 (68.6%)
	1		0	7 (38.9%)	0	0	38 (31.4%)
	*Total*		1	18	1	6	121
Mesial accessory cusp	0		**1 (100%)**	13 (81.2%)		5 (83.3%)	71 (61.2%)
	1	x	0	3 (18.8%)		1 (16.7%)	45 (38.8%)
	*Total*		1	16		6	116
Source			a,b	a,b	c^b^	b,d	a,b

*Note:* (a) Martinón‐Torres et al. (2012, table 11); (b) Bailey (2002, table 5.2); (c) Hublin et al. (2012), Dar es Soltane II (DS6) and Contrebandiers (T5); (d) Hershkovitz et al. (2018), Misliya.

^a^ In Bailey (2002) distal and mesial accessory ridges were pooled and not reported here.

^b^ Supplemented with our own observations.

#### 3.1.4 Comparative Morphological Assessment

The above‐described crown morphologies were compared to published data on non‐metric dental traits (see SOM Tables S1 and 2, 3, 4). Although there is often overlap between groups in the expression of non‐metric dental features, their combination and frequencies can be diagnostic (e.g., Bailey 2002, 2006; Martinón‐Torres et al. 2012).

In the UC, the combination of weak shoveling, a weakly developed *tuberculum dental*e, and a weakly expressed mesial marginal ridge is more typical for early and later *H. sapiens* . In contrast, Neanderthals and Middle Pleistocene individuals from Europe tend to exhibit a more pronounced expression of these features (Bailey 2006; Martinón‐Torres et al. 2012; Table 2). Differences in the way grades for distal accessory ridges are reported in the literature complicate their evaluation in a comparative framework. For example, some studies pool the results in absence vs. presence, while others break down the presence category into several grades. El'Aliya shows a weak distal accessory ridge (grade 1, cf. SOM Table S1). Distal accessory ridges of similar manifestation are found in most of our comparative groups (Table 2). The reported pooled grades 1–5 do not allow for differentiating between weak and strong expressions of this trait in early *H. sapiens* (cf. Bailey 2006). However, early *H. sapiens* are the only group in which the distal accessory ridge is typically present (Table 2).

Most UP3 traits do not place el'Aliya clearly within any of the comparative samples considered here due to the substantial overlap between groups (Table 3). However, its non‐bifurcated essential crests align it best with early and later *H. sapiens* (Bailey 2006; Martinón‐Torres et al. 2012). The presence of a distal accessory ridge and absence of a mesial one on the el'Aliya UP3 paracone are difficult to interpret within our comparative samples as these features are usually pooled in published data (e.g., Bailey 2006). Pooled samples show low‐to‐medium frequencies of accessory ridges on the UP3 paracone in all later *Homo* groups. Dental wear precluded the evaluation of this trait in our Aterian sample (cf. Hublin et al. 2012, Table 3).

In contrast for the UP4, the presence of premolar accessory ridges is observed in higher frequencies in Middle Pleistocene Europeans and Neanderthals than in *H. sapiens* (Bailey 2006; Liao et al. 2019; Martinón‐Torres et al. 2012; Table 4). However, this trait is present in el'Aliya and another Aterian (Contrebandiers T5) individual from Morocco, as well as in the Skhul/Qafzeh sample (cf. Hublin et al. 2012, Table 4). Further, the absence of bifurcated essential crests groups the el'Aliya UP4 with early and later *H. sapiens* and separates it from Neanderthals and Middle Pleistocene Europeans (Bailey 2006; Martinón‐Torres et al. 2012).

In summary, the el'Aliya dentition, and especially the UC, is most similar to early and later *H. sapiens* in its combinations of most non‐metric traits (cf. Bailey 2006; Hublin et al. 2012; Martinón‐Torres et al. 2012; Liao et al. 2019; Tables 2, 3, 4).

### 3.2 External Crown Dimensions

**FIGURE 3:**
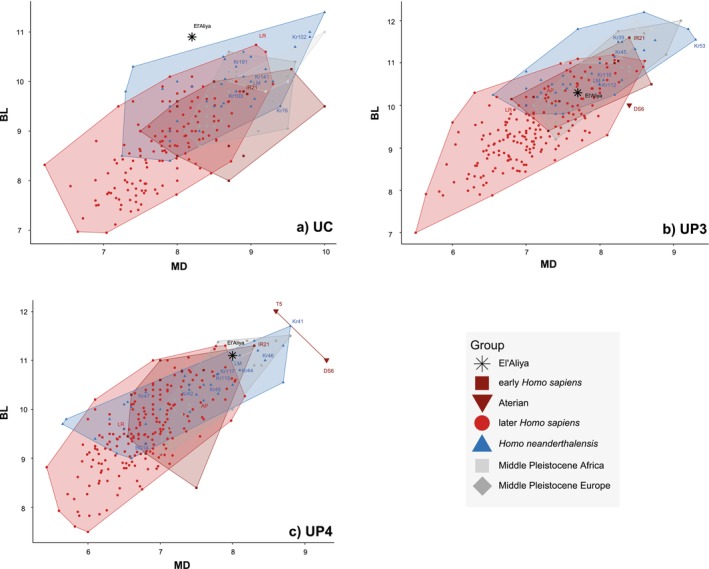
Bivariate plot of crown diameters in mm of (a) upper permanent canines (UC), (b) third (UP3) and (c) fourth premolars (UP4). Detailed information about the sample is provided in SOM Table S3 and summary statistics in SOM Table S7. Abbreviations as in Table 1. BL = buccolingual; MD = mesiodistal.

Dental crown dimensions for UCs, UP3s, and UP4s overlap greatly between our comparative samples (Figure 3; SOM Table S7). The el'Aliya UC MD crown dimension (8.2 mm) falls between the means of Neanderthals, early and later *H. sapiens* , whereas its BL dimension (10.9 mm) is one of the largest in our sample (Figure 3a; SOM Table S7). Only three early Neanderthal UCs from Krapina (37, 102, and 144) and the Broken Hill (Kabwe) specimen show slightly larger BL diameters. The diameters of the el'Aliya UP3 (MD 7.7 mm, BL 10.3 mm) are most similar to the mean of early *H. sapiens* and fall within the range of most of the comparative groups (Figure 3b). In contrast, the values for the UP4 (MD 8 mm, BL 11.1 mm) are most similar to the mean of Middle Pleistocene individuals from NW Africa (SOM Table S7). Most Middle Pleistocene individuals in our sample, including Neanderthals from Krapina and Jebel Irhoud 21, show similarly large or larger UP4s. The specimens with even higher levels of megadonty are two Aterian teeth from the region of Témara, Morocco (Hublin et al. 2012; Figure 3c).

The megadonty observed in the Aterian individuals and teeth from el'Aliya is reflected in their estimated crown area (MD*BL) (SOM Figure S4). For the UC, only African Middle Pleistocene individuals show, on average, greater areas. The el'Aliya UP4 plots close to the mean of Middle Pleistocene Africans, while the Aterian specimens are even larger. In contrast, el'Aliya and other Aterian UP3s fall in the overlap of early *H. sapiens* , Neanderthals, and Middle Pleistocene Europeans.

### 3.3 EDJ Analyses

#### 3.3.1 UC: EDJ Morphology

**FIGURE 4:**
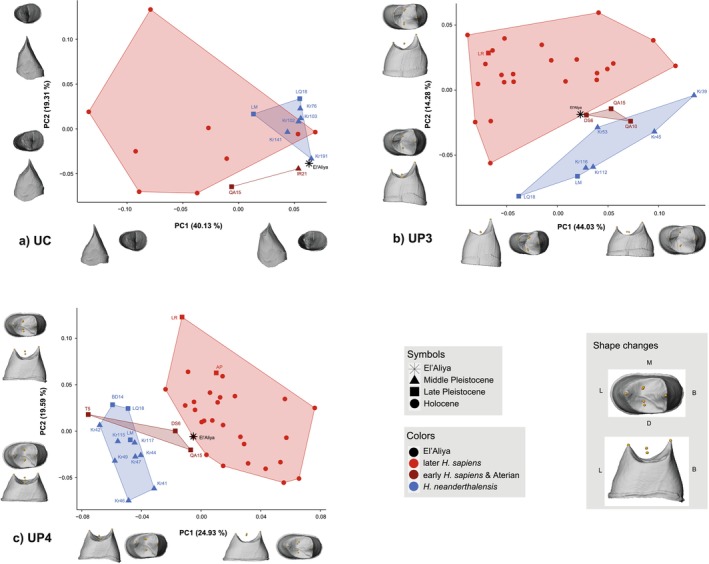
EDJ shape PCAs of (a) upper permanent canines (UC), (b) third (UP3) and (c) fourth premolars (UP4) with Mugharet el'Aliya and all fossil *H. sapiens* projected into the plots of PC1 against PC2. Shape changes along PCs are illustrated as deformations at ±2 sd. Information about the sample and abbreviations of all fossil individuals are listed in Table 1.

Neanderthal and *H. sapiens* UC shapes overlap substantially along PCs 1 and 2. The el'Aliya UC plots outside all convex hulls, falling between early *H. sapiens* and Neanderthals, and closest to a canine from Krapina (Figure 4a; cf. PD results). Our early *H. sapiens* sample is separated from both later *H. sapiens* and Neanderthals. PC1 (40.13% of variance) summarizes shape changes from a relatively oval outline with a more lingually positioned cusp, and a higher dentine body coupled with a lower dentine horn (more positive values) to a relatively triangular outline with a more buccally positioned cusp, and a lower dentine body coupled with a higher dentine horn (more negative values). PC2 (19.31% of variance) captures intra‐specific variation (Figure 4a) and reflects shape changes from a relatively narrower, shorter, and higher crown with an expanded buccal surface (more positive PC2 scores) to a relatively longer, wider, and lower crown with a reduced buccal surface (more negative PC2 scores).

Allometry was detected both in a weak positive correlation between overall shape, in the form of Procrustes coordinates, and log CS (SOM Table S8) and in a strong positive correlation between log CS and PC1 (Pearson correlation test: *t* = 3.195, DoF = 17, *R* = 0.606, *p* = 0.006). Our later *H. sapiens* individuals exhibit a greater intra‐specific variation along PC1 than predicted by their size, while the, on average, larger fossil individuals, including el'Aliya, predominantly show positive PC1 scores (SOM Figure S5).

The UC of el'Aliya is closest in its overall shape (pairwise PD distances) to two Neanderthal individuals from Krapina.

#### 3.3.2 UP3: EDJ Morphology

The PCA of UP3 shape separates *H. sapiens* and Neanderthals on a combination of PC1 and PC2 (Figure 4b). El'Aliya falls within the convex hull of later *H. sapiens* and close to that of our early *H. sapiens* sample. The early *H. sapiens* sample plots intermediate between later *H. sapiens* and Neanderthals, with some overlap with the Neanderthals. PC1 (44.03% of variance) summarizes shape changes from relatively wider, longer, and lower crowns with a slightly higher paracone than the protocone (more positive values) to relatively narrower, shorter, and higher crowns with a substantially higher paracone (more negative values). PC2 (14.28% of variance) reflects shape changes from relatively longer crowns with a buccally lower and narrower dentine body, a mesial marginal ridge of equal height to the distal one, and increased enamel thickness (more positive values) to relatively shorter crowns with a buccally higher and wider dentine body, a taller mesial than distal marginal ridge, and decreased enamel thickness (more negative values).

Allometry was detected in the form of a moderate negative correlation between log CS and PC2 (Pearson correlation test: *T* = −3.365, DoF = 33, *R* = −0.500, *p* = 0.002). Along PC2, Neanderthal UP3s, which tend to be larger, show negative values, while most UP3s from our later *H. sapiens* sample, which tend to be smaller, exhibit positive values (cf. Figure 4b; SOM Table S7). When plotting PC2 versus log CS, the early *H. sapiens* specimens almost completely overlap with Neanderthals, and el'Aliya plots outside all convex hulls but intermediate between early and later *H. sapiens* (SOM Figure S6).

The el'Aliya ULP3 is most similar in its overall shape, as reflected by PD, to three Holocene North African later *H. sapiens* from our sample.

#### 3.3.3 UP4: EDJ Morphology

Overall, results for the el'Aliya ULP4 are similar to those from the ULP3. The el'Aliya UP4 plots just outside the later *H. sapiens* convex hull and close to our early *H. sapiens* sample, which overlaps with the Neanderthals (Figure 4c). PC1 (24.93% of variance) reflects shape changes from relatively longer crowns with buccally lower dentine bodies, mesial and distal marginal ridges of equal height, and increased enamel thickness (more positive scores) to relatively shorter crowns with buccally higher dentine bodies, a taller mesial marginal ridge relative to the distal one, and decreased enamel thickness (more negative scores). PC2 (19.59% of variance) summarizes shape changes from a relatively shorter and narrower crown with a higher dentine body, increased enamel thickness, slightly taller paracone relative to the protocone, and mesial and distal marginal ridges of equal height (more positive scores) to a relatively longer and wider crown with a lower dentine body, decreased enamel thickness, paracone and protocone of equal height, and a taller mesial marginal ridge relative to the distal one (more negative scores) (Figure 4c).

Allometry was detected in a strong negative correlation between log CS and PC1 (Pearson correlation test: *T* = 5.965, DoF = 42, *R* = ‐0.678, *p* ≤ 0.01). PC1 separates the, on average, smaller later *H. sapiens* teeth (more positive values) from larger Neanderthals (more negative values; cf. Figure 4c; SOM Table S7). When plotting PC1 against log CS, el'Aliya plots between the early and later *H. sapiens* convex hulls, while early *H. sapiens* overlap with the Neanderthals (SOM Figure S7).

The el'Aliya ULP4 is most similar in its overall shape (pairwise PD distances) to a Holocene North African later *H. sapiens* , closely followed by two Holocene European later *H. sapiens* .

### 3.4 Perikymata Analysis UC

**FIGURE 5:**
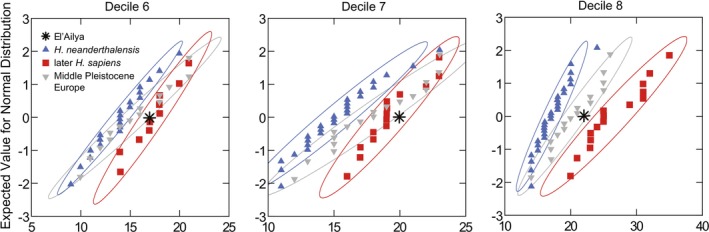
Probability plots of perikymata counts per decile for upper canines (UC). Visualization of perikymata counts on the *x*‐axis against the expected value for a normal distribution on the *y*‐axis. Detailed information about the sample and underlying perikymata counts in SOM Tables S6 and S9, respectively. 90% confidence ellipses shown.

The analysis of the PPP in the el'Aliya UC was restricted to deciles 5, 6, 7, and 8 (SOM Table S9), because a clear visualization of the perikymata in all other deciles was difficult due to poor preservation and the use of curation materials on the tooth. In decile 5, the perikymata count in the UC overlaps largely between *Neanderthals* and later *H. sapiens* from the European Upper Paleolithic (Ramirez Rozzi and Bermúdez de Castro 2004). For this reason, decile 5 was not considered for the calculation of probability plots. The number of perikymata in the examined deciles of the ULC from el'Aliya falls within one standard deviation of the mean perikymata counts for *H. sapiens* and outside that for Neanderthals. In the corresponding probability plots, the el'Aliya UC also falls outside the 90% confidence interval of Neanderthals for all deciles (Figure 5). In contrast, el'Aliya plots inside the 90% confidence interval of later *H. sapiens* in deciles 6 and 7 and that of European Middle Pleistocene individuals in decile 8. In deciles 6 and 7, el'Aliya falls right outside the confidence interval of Middle Pleistocene Europeans.

## 4 Discussion

### 4.1 Morphological Affinities

Despite its exceptional size, the el'Aliya dentition shows a relatively simple crown morphology, aligning it with early and later *H. sapiens* (Martinón‐Torres et al. 2012; Bailey 2006; Tables 2, 3, 4). Neanderthals and Middle Pleistocene Europeans tend to have a more complex crown morphology, including a more pronounced expression of the tuberculum dentale, UC shoveling, and bifurcated premolar essential crests (Martinón‐Torres et al. 2012; Bailey 2006).

The external crown morphology can be altered by causes ranging from tooth wear to taphonomy. The internal tooth morphology of the EDJ is phylogenetically informative (e.g., Skinner and Kapadia 2005; Skinner et al. 2008; Pan et al. 2016; Zanolli et al. 2022) and allows the inclusion of comparatively larger samples (e.g., including worn crowns with an intact EDJ). El'Aliya's canine EDJ morphology is similar to that of Neanderthals (Figure 4a); however, so is that of the early *H. sapiens* individual Irhoud 21. Both the Aterian and Jebel Irhoud fossils are known to show a mixture of primitive and derived traits (e.g., Ferembach 1976; Hublin and Tillier 1981; Smith et al. 2007; Hublin et al. 2012, 2017; Bergmann et al. 2022; Bailey et al. 2017; Harvati and Hublin 2012). The origin of these primitive traits in the Aterian fossil record remains debated, with the retention of ancestral traits due to regional continuity being one of the main hypotheses discussed (e.g., Hublin 2000; Hublin et al. 2017; Nespoulet et al. 2008; Bailey et al. 2017; Harvati and Hublin 2012; Bergmann et al. 2022; Röding et al. 2023). It must be noted that (1) our UC samples are very small, and (2) the Neanderthals overlap considerably with the later *H. sapiens* . Due to these sample restrictions, it remains inconclusive whether other processes, like the retention of ancestral features, play a role in addition to the observed allometry in UCs. In contrast, the EDJ shapes of the premolars are most similar to *H. sapiens*. Both premolar EDJ analyses separated later *H. sapiens* from Neanderthal samples, with early *H. sapiens* plotting intermediate and overlapping partially with Neanderthals (Figure 4b,c). Despite their exceptional size, the el'Aliya premolar shapes plot within the *H. sapiens* variation. Nevertheless, the temporal trend for reduction in dental crown dimensions from the Middle Pleistocene to the Holocene (SOM Table S7; Brace et al. 1987; Gómez‐Robles et al. 2017) is reflected in the PCA plots due to the allometric relationships between log CS and PC2 and PC1 in UP3s and UP4s, respectively. Further, the PCA results (e.g., Figure 4a PC1) follow the expectation of relatively thinner enamel in Neanderthals (e.g., Martín‐Francés et al. 2023; Olejniczak et al. 2008; Buti et al. 2017). The inclusion of landmarks indirectly capturing enamel thickness played an important role in separating our comparative groups and thereby, in grouping el'Aliya with our *H. sapiens* sample.

Additionally, the el'Aliya UC displays a PPP most similar to *H. sapiens* (Figure 5; SOM Table S9). The principal differences in the PPP of the UC between Neanderthals and later *H. sapiens* from the European Upper Paleolithic reside in deciles 8, 9, and 10 and to a lesser degree in deciles 6 and 7 (Ramirez Rozzi and Bermúdez de Castro 2004). In the perikymata counts per decile, Neanderthals show lower mean values. The perikymata counts for the el'Aliya canine fall close to the *H. sapiens* mean except for decile 8, where the count is at the lower *H. sapiens* range (SOM Table S9; Figure 5). Based on the similarity of the PPP to *H. sapiens* , we might infer a *H. sapiens* ‐like crown formation (Ramirez Rozzi and Bermúdez de Castro 2004).

### 4.2 Possible Hybridization Effects

El'Aliya exhibits two morphological traits that warrant further discussion: (1) The unexpectedly large size of its canine and UP4 (Figure 3a,c), and (2) its “Neanderthal”‐like canine EDJ shape (Figure 4a) are at odds with a *H. sapiens* attribution. One might hypothesize admixture due to previous interpretations of atypical combinations of *H. sapiens* ‐like and Neanderthal‐like features, including large size, as potential evidence for admixture (see Ackermann 2011; Harvati et al. 2007). Hybridization between Neanderthals and *H. sapiens* has been documented in several instances through paleogenetic analyses (see e.g., Green et al. 2010; Fu et al. 2016; Hajdinjak et al. 2021; Posth et al. 2017; Villanea and Schraiber 2019; Peyrégne et al. 2022). Indeed, all Upper Paleolithic genomes sequenced to date show evidence of contributions from Neanderthals (see e.g., Bergström et al. 2021; Harvati and Ackermann 2022). Interbreeding has also been suggested for other archaic lineages, including between early *H. sapiens* and Denisovans, Denisovans and Neanderthals, as well as early *H. sapiens* and “ghost” lineages in Africa (e.g., Browning et al. 2018; Reich et al. 2011; Durvasula and Sankararaman 2020; Hsieh et al. 2016; Lachance et al. 2012; Slon et al. 2018). Previous interpretations of fossil dental samples, including those from La Cotte de St. Brelade, Jersey (Compton et al. 2021), and Bacho Kiro, Bulgaria (Hublin et al. 2020), have attributed large size as well as the mosaic of Neanderthal and *H. sapiens* features as evidence supporting hybridization. This interpretation was supported by subsequent ancient DNA analyses for the Bacho Kiro individual (Hajdinjak et al. 2021). Furthermore, model organism studies (based on e.g., mice and tamarins) indicate that unexpectedly large canine size might be a side effect of hybridization (e.g., Cheverud et al. 1993; Percival et al. 2016; Leamy and Thorpe 1984). However, the study of other model organisms (e.g., moles) showed that relatively high frequencies of dental anomalies might be caused by genetic drift, especially in marginal populations (e.g., Asahara et al. 2012; Kryštufek, Spitzenberger, and Kefelioğlu 2001).

Given the geographic position of North Africa as a potential dispersal corridor of Pleistocene hominins across Africa and Europe (e.g., Reyes‐Centeno et al. 2017; Beyer et al. 2021), the combination of features observed in el'Aliya may suggest potential admixture. Nevertheless, it is important to note that the observed morphology could also be the result of other evolutionary processes, including, for example, retention of ancestral traits, adaptation to local environments, and genetic drift in marginal populations. In other words, the problem of equifinality must always be kept in mind when assessing the potential impact of hybridization (Harvati and Ackermann 2022). This hypothesis, therefore, cannot be resolved here but should be evaluated further with paleogenetic data and in the context of increased understanding of the variation of the NW African fossil record, if possible.

### 4.3 Mugharet el'Aliya and the Aterian

The Aterian fossil record consists exclusively of Moroccan material, and remains are poorly described or even unpublished (e.g., Hublin et al. 2012; Freidline et al. 2024). The results of our analysis of the el'Aliya fossil can improve our understanding by adding the first comparative analysis of an UC and information about additional upper premolars as well as their variation within the Aterian. Both el'Aliya premolars exhibit an extreme mesial displacement of the protocone that exceeds the displacement observed in all other individuals included in our datasets (cf. Figure 1). The two Aterian individuals from Dar‐es‐Soltane (DS6) and Contrebandiers (T5) also show a mesial displacement of the protocone in their premolars, but less extreme (Hublin et al. 2012). Mesially displaced protocones in upper premolars of varying degrees have been reported for many hominin groups, including *H. sapiens* , Neanderthals, and European Middle Pleistocene individuals (e.g., Martinón‐Torres et al. 2019; Gómez‐Robles et al. 2011). Furthermore, the crown dimensions of the el'Aliya UP3 show a less pronounced level of megadonty than in the other tooth types and other Aterian individuals (Figure 3; SOM Figure S4; Hublin et al. 2012). However, crown dimensions are only available for one larger additional UP3 associated with the Aterian, Dar‐es‐Soltane (DS6). Therefore, it remains unclear if the less pronounced level of megadonty in the el'Aliya UP3 is atypical for the Aterian.

Nevertheless, el'Aliya follows the dental pattern described for other individuals associated with the Aterian where the combination of crown dimensions and non‐metric traits distinguishes them from Neanderthals and best aligns them with *H. sapiens* (Hublin et al. 2012; Bailey et al. 2017). The el'Aliya canine and fourth premolar share megadonty with the Aterian fossils while exhibiting relatively simple external crown morphology (Figure 3a,c, Tables 2 and 4; e.g., Ferembach 1976; Hublin and Tillier 1981; Smith et al. 2007; Hublin et al. 2012). The external crown morphology is more complex in other Aterian teeth, especially molars, due to mass‐additive traits (Hublin et al. 2012). Nevertheless, the premolars from Dar‐es‐Soltane (DS6) and el'Aliya share features like the presence of accessory ridges in the UP4 and an increase in crown area from the third to the fourth premolar (Table 3, SOM Figure S4; Hublin et al. 2012). The latter is also shared with some individuals from Qafzeh (Hublin et al. 2012). Thereby, our results add to the growing body of reported morphological similarities of the face and the dentition between Late Pleistocene individuals from North Africa and Near Eastern samples from Qafzeh and Skhul (e.g., Harvati and Hublin 2012; Hublin et al. 2012; Hublin and Tillier 1988; Röding et al. 2023; Freidline et al. 2024). Further, all early *H. sapiens* in our sample, including individuals associated with the Aterian and those from Qafzeh, showed similarities in overall premolar EDJ shape based on Procrustes distances. Nevertheless, the two fourth premolars associated with the Aterian (DS6, T5) exhibit a relatively large variation regarding shape captured along PC1 of EDJ shape space (Figure 4c).

Overall, our results add to the evidence supporting regional continuity in the hominin fossil record associated with the North African MSA (Hublin 2000; Hublin et al. 2017; Nespoulet et al. 2008; Bergmann et al. 2022; Freidline et al. 2024). The fossils associated with the Aterian from Dar‐es‐Soltane (DS6) and Contrebandiers (T5) even if at least 20–30 ka older than el'Aliya (Hublin et al. 2012) still appear similar in overall shape, crown dimensions, and crown non‐metric traits (cf. Figure 4, Tables 3 and 4, SOM Figure S4, and SOM Table S7). In contrast, the Moroccan Later Stone Age (LSA) sample of Iberomaurusians shows significantly reduced crown dimensions for all three tooth types (e.g., Voisin et al. 2012; Caillard 1978). This supports the argument for regional continuity in the MSA hominin fossil record and a discontinuity with later populations associated with the LSA (Scerri 2017; Hublin 2000; Hublin et al. 2017; Nespoulet et al. 2008; Harvati and Hublin 2012). Our results do not support the suggestion that regional continuity extended into the LSA, as recently proposed based on a re‐evaluation of, among others, North African mandibular morphology from the later Early Pleistocene to the Holocene (Bergmann et al. 2022).

### 4.4 Methodological Considerations

To our knowledge, this study is one of the first to propose a landmark set for the study of the UC EDJ (cf. Figure 2b; SOM Table S5). This landmark set captures the tendency of *H. sapiens* UCs to appear slender compared to relatively more robust UCs in Neanderthals (Figure 4a). Further, the on average larger relative BL dimension (BL/MD) in Neanderthal UCs is reflected in shape PC1, with Neanderthals showing more BL elongated oval shapes compared to most *H. sapiens* UCs (cf. SOM Table S7). However, due to the limited number of available fixed landmarks on the EDJ in combination with a high but not perfect correlation between the EDJ and external crown features (cf. Skinner et al. 2010; Morita 2016), the landmark dataset proposed here does not capture many shape aspects commonly used to differentiate between Neanderthals and *H. sapiens* (cf. Table 2; Martinón‐Torres et al. 2012, Bailey 2006). To adequately incorporate the EDJ equivalent of non‐metric crown features, like shoveling and the tuberculum dentale, would require the addition of surface semi‐landmarks (e.g., Gunz et al. 2005) or the use of alternative methodologies like the surface registration method (e.g., Röding et al. 2023).

## 5 Conclusions

Our previous analysis of Mugharet el'Aliya's maxilla found early *H. sapiens* affinities for this specimen, suggesting that its “archaic” morphology was mainly related to its large size (Röding et al. 2023). The analysis of this individual's dentition supports this attribution by showing a similar mosaic between morphology and size as the maxilla. The external and internal dental morphology show the greatest affinities to early and later *H. sapiens*. The megadont canine and fourth premolar, while unusual for *H. sapiens* , are typical for the Aterian. Therefore, our results conform with Mugharet el'Aliya representing an Aterian juvenile *H. sapiens* individual. The analyses of the previously unpublished Mughaet el'Aliya UP4 add to our knowledge of variation in Aterian premolars. It is essential to understand the variation in the Aterian fossil record to build a base for deciphering the evolutionary processes underlying modern human origins in the region.

## Author Contributions

**Carolin Röding:** conceptualization (equal), data curation (lead), formal analysis (lead), investigation (lead), methodology (lead), resources (equal), software (lead), validation (lead), visualization (lead), writing – original draft (lead), writing – review and editing (equal). **Sireen El‐Zaatari:** formal analysis (supporting), funding acquisition (supporting), investigation (supporting), methodology (supporting), resources (equal), writing – review and editing (equal). **Fernando V. Ramirez Rozzi:** data curation (supporting), formal analysis (supporting), funding acquisition (supporting), investigation (supporting), methodology (supporting), resources (equal), software (supporting), visualization (supporting), writing – review and editing (equal). **Chris Stringer:** conceptualization (equal), funding acquisition (supporting), writing – review and editing (equal). **M. Loring Burgess:** data curation (supporting), resources (equal), writing – review and editing (equal). **Rodrigo S. Lacruz:** conceptualization (equal), funding acquisition (supporting), resources (equal), writing – review and editing (equal). **Katerina Harvati:** conceptualization (equal), funding acquisition (lead), resources (equal), supervision (equal), writing – original draft (supporting), writing – review and editing (equal).

## Ethics Statement

We only employed non‐destructive techniques (literature review, Computer Tomography scans and high‐resolution dental casts). Although part of our Computer Tomography sample is labeled as Holocene, these samples pre‐date 1500 AD, and we can exclude the existence of living relatives with less than 10 generations of separation. Further, with the exception of the fossil in focus, that is, Mugharet el'Aliya, we did not handle the actual human remains. Our comparative samples exclusively consisted of already existing data either in the form of (1) raw data extracted from the literature, or (2) digital data and high‐resolution casts that were included in previous publications. For information about institutional policies about repatriation please see the respective institutions' web pages.

## Conflicts of Interest

The authors declare no conflicts of interest.

## Supporting information


**Data S1.** Supporting Information.

## Data Availability

The raw data for dental crown dimensions presented in the current study are available from ZENODO, including the recent *H. sapiens* data provided by S.E.B. The non‐metric data for Mugharet el'Aliya is presented in Tables 2, 3, 4 and references for the published comparative data are provided. EDJ data are available from C.R. upon request pending permission from the individual repositories. Perikymata data for Mugharet el'Aliya are shown in SOM Table S9, and comparative data are available from F.R.R. upon reasonable request.
