# Supplementary material for: Dentition of the Mugharet El'Aliya Fossil Human Maxilla, Morocco
Source: Am J Biol Anthropol. 2025 Feb 22;186(2):e70015. doi: 10.1002/ajpa.70015 (PMC11845900; doi:10.1002/ajpa.70015)
Supplement: Supplementary file 1 — Data S1. Supporting Information. [file AJPA-186-e70015-s001.pdf]

Supplementary Online Material (SOM):

Dentition of the Mugharet el'Aliya fossil human maxilla, Morocco

Supplementary Figures 2
SOM Figure S1 3
SOM Figure S2 4
SOM Figure S3 5
SOM Figure S4 6
SOM Figure S5 7
SOM Figure S6 8
SOM Figure S7 9
Supplementary Tables 10
SOM Table S1: 11
SOM Table S2: 12
SOM Table S3 13
SOM Table S4 14
SOM Table S5 15
SOM Table S6 16
SOM Table S7: 17
SOM Table S8 18
SOM Table S9 19
References 20

Supplementary Figures

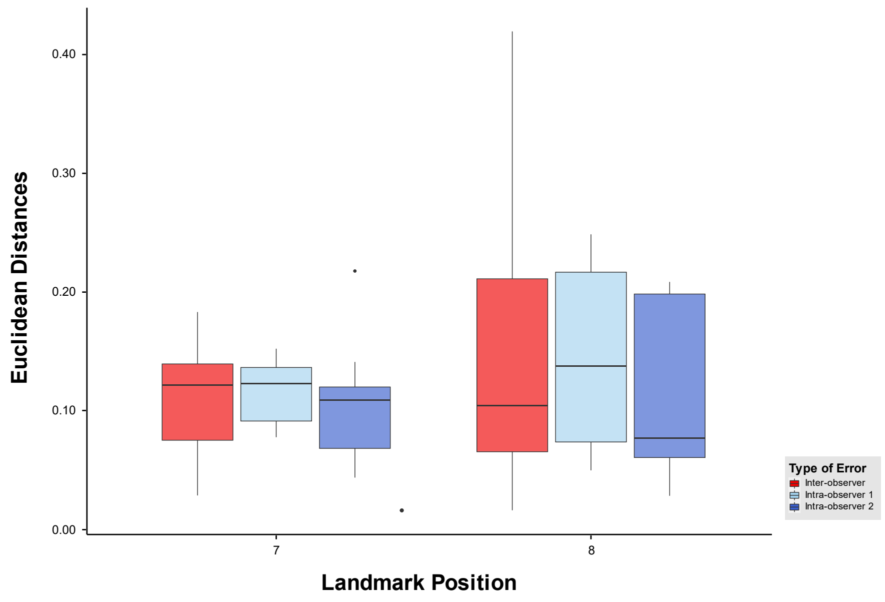

SOM Figure S1: Visualization of intra- and inter-observer error for the UC EDJ dataset. Euclidean distances (ED) are calculated in mm between repeated measurements of the same landmark position. Detailed landmarks definitions are listed in SOM Table S5 and their visualization is shown in Figure 2b.

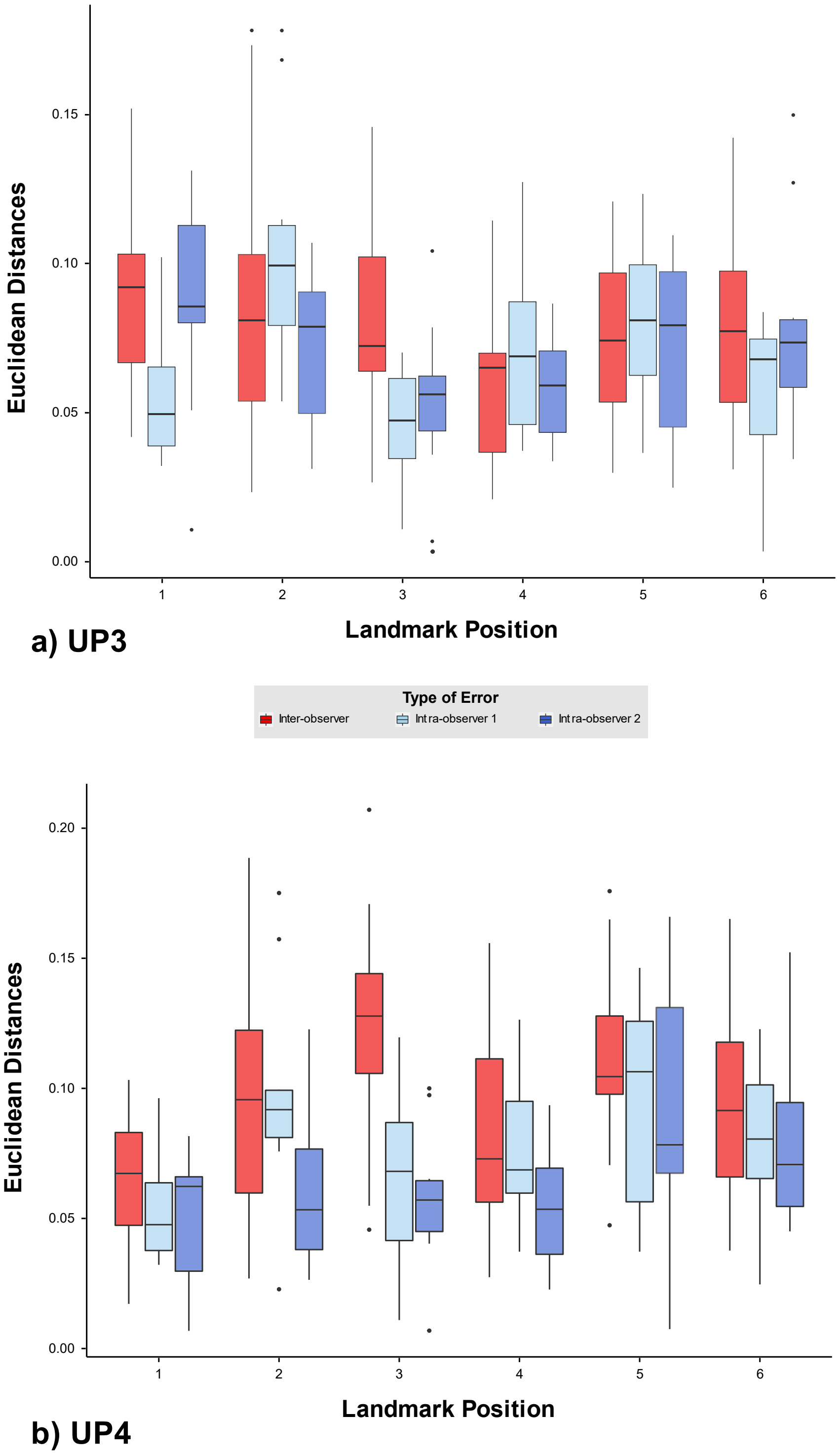

SOM Figure S2: Visualization of intra- and inter-observer error for the a) UP3 and b) UP4 EDJ datasets. Euclidean distances (ED) are calculated in mm between repeated measurements of the same landmark position. Detailed landmarks definitions are listed in SOM Table S5 and their visualization is shown in Figure 2a.

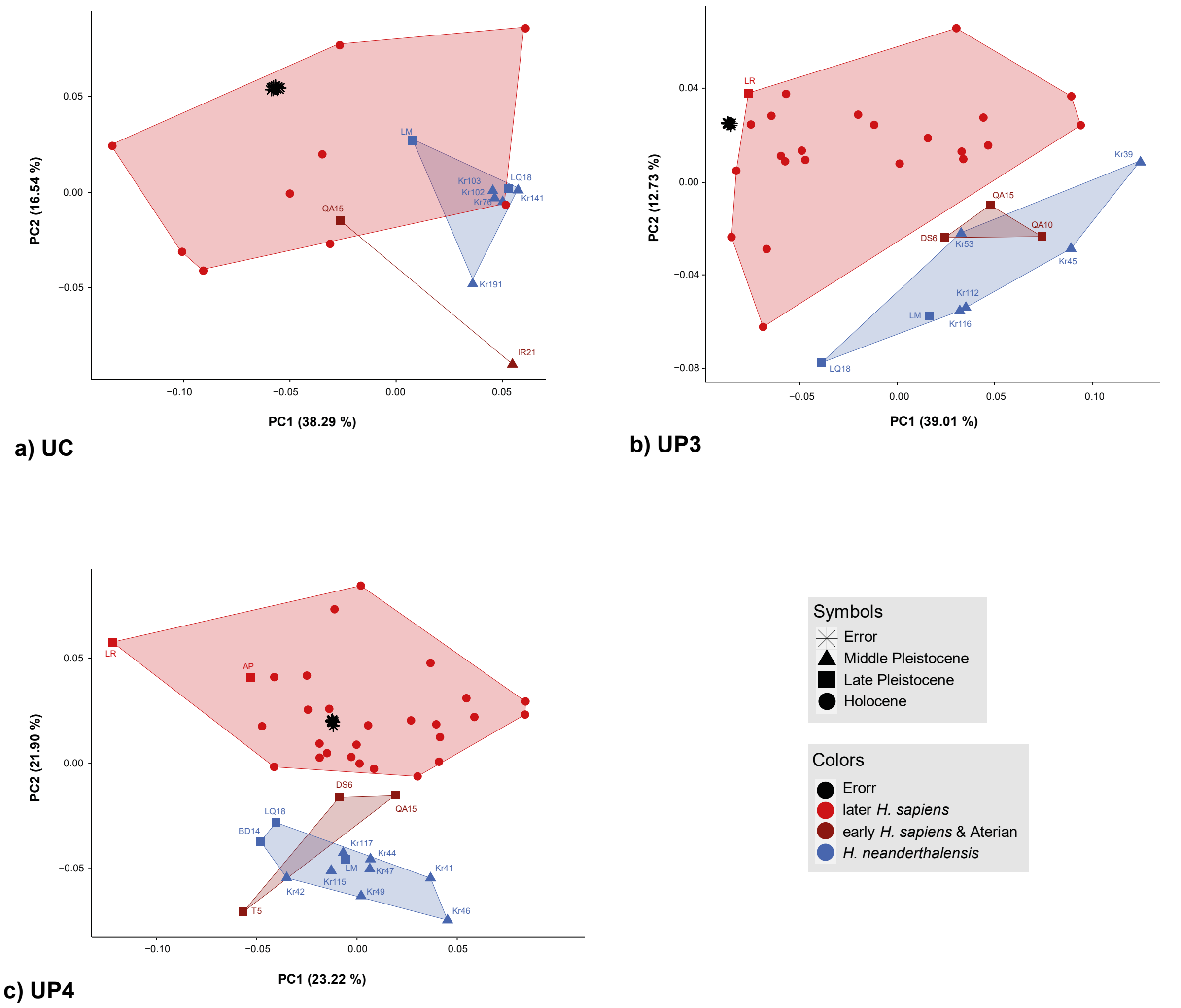

SOM Figure S3: EDJ shape PCAs of a) upper permanent canines (UC), b) third (UP3), and c) fourth premolars (UP4) with all repeated error measurements projected into the plots of PC1 against PC2. Abbreviations as in SOM Table S4.

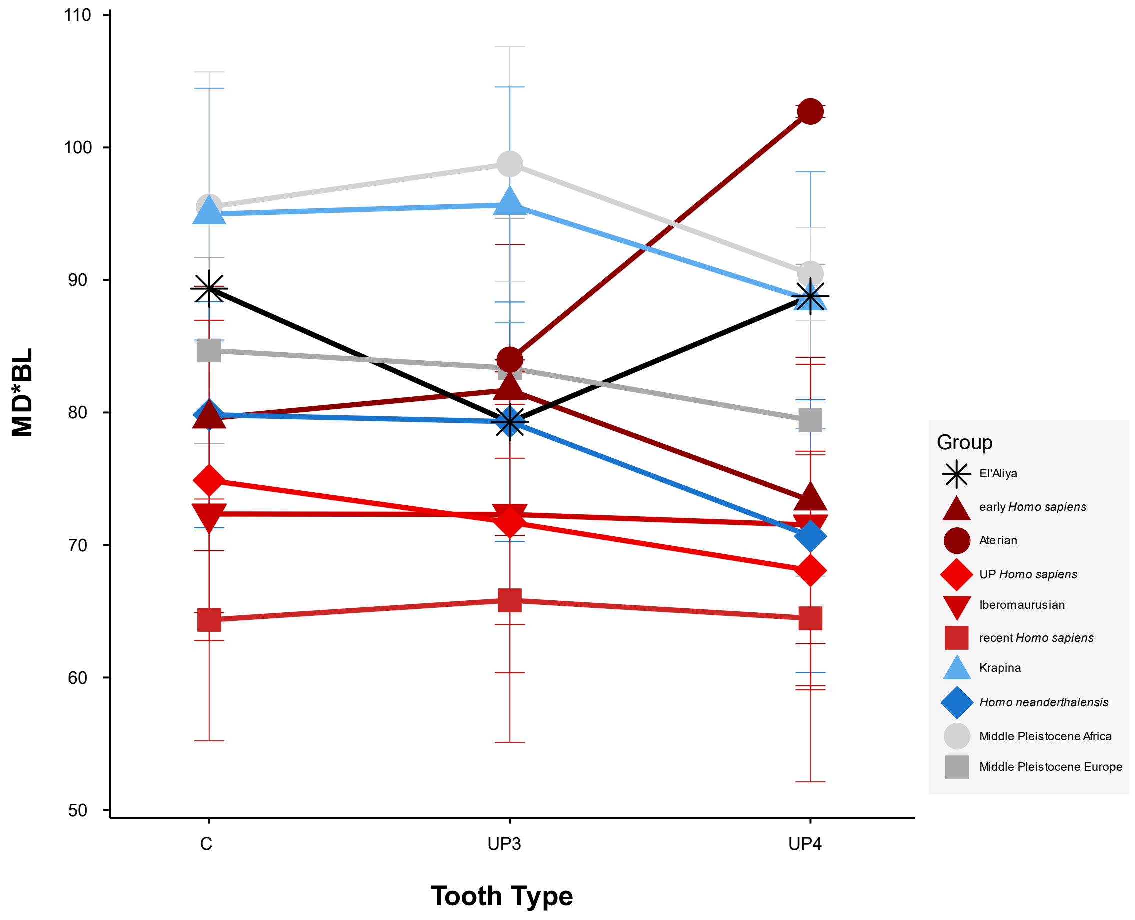

SOM Figure S4: Visualization of crown area (MD*BL) per tooth type. Crown area shown in mm^2 and tooth types from left to right: UC, UP3 and UP4. Group mean values are illustrated as symbols and standard deviations (sd) as range. Detailed information about the sample in SOM Table S3 and underlying values in SOM Table S7.

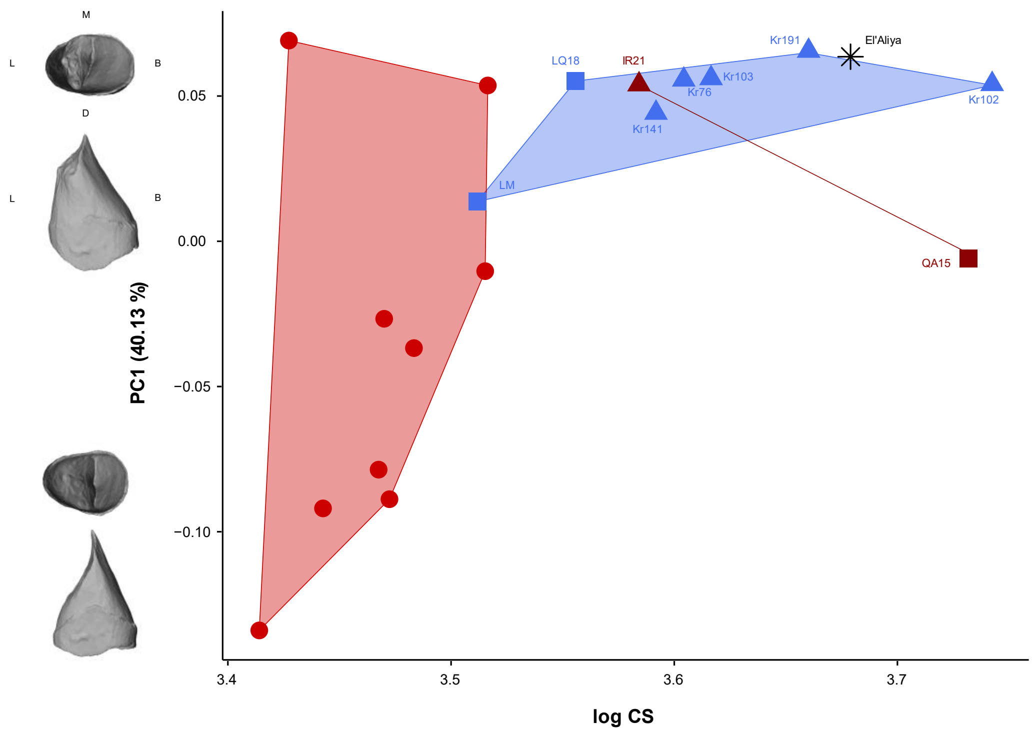

SOM Figure S5: Upper permanent canine (UC) shape PCA with Mugharet el'Aliya and all early *H. sapiens* fossils projected into the plot. PC1 plotted against log CS. Symbols, colors, and abbreviations as in Figure 4 and SOM Table S4.

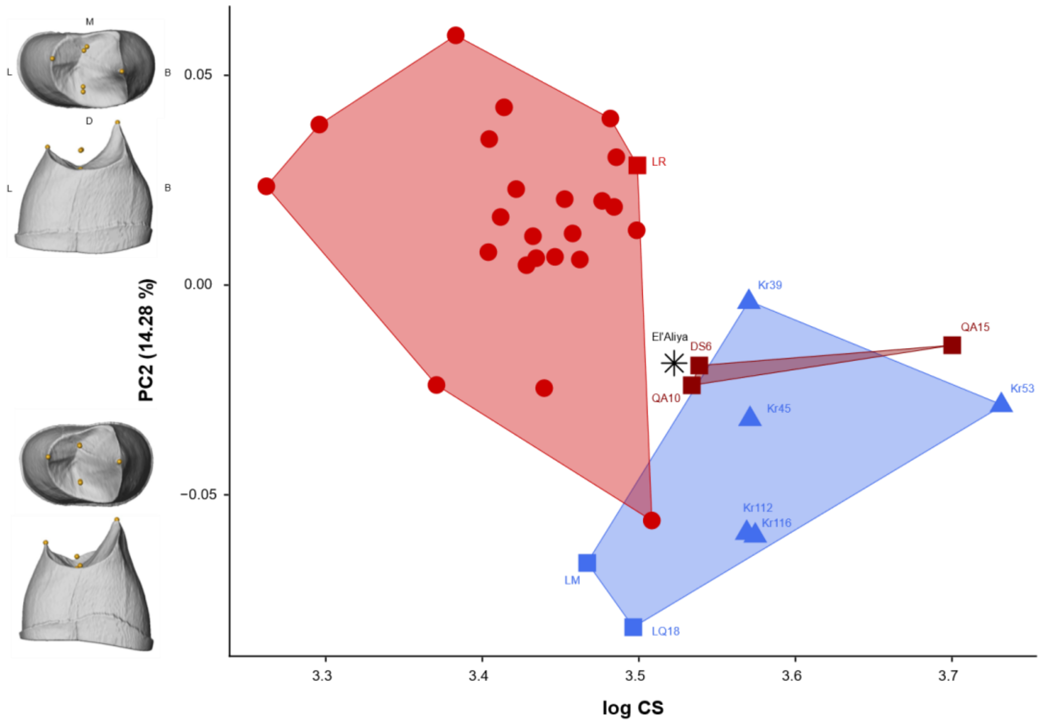

SOM Figure S6: Upper permanent third premolar (UP3) shape PCA with Mugharet el'Aliya and all early *H. sapiens* fossils projected into the plot. PC2 plotted against log CS. Symbols, colors, and abbreviations as in Figure 4 and SOM Table S4.

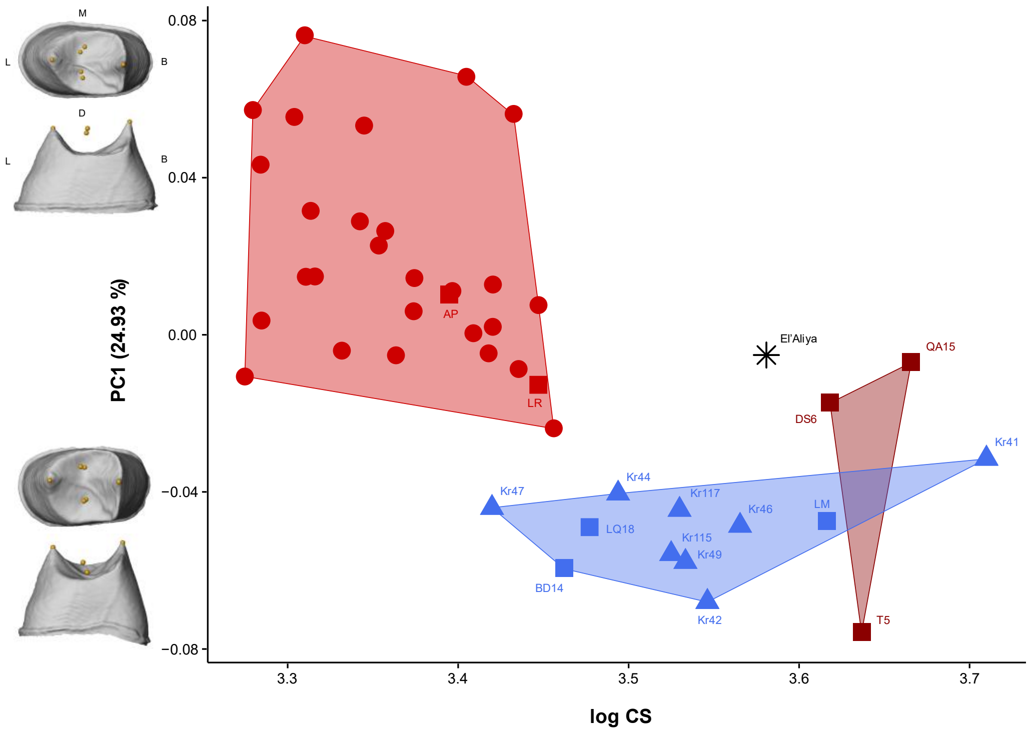

SOM Figure S7: Upper permanent fourth premolar (UP4) shape PCA with Mugharet el'Aliya and all early *H. sapiens* fossils projected into the plot. PC1 plotted against log CS. Symbols, colors, and abbreviations as in Figure 4 and SOM Table S4.

Supplementary Tables

SOM Table S1: Description of non-metric traits used for the comparison of upper permanent canines (UC), third premolars (UP3), and fourth premolars (UP4). Definitions, grades, and their descriptions are based on Martinón-Torres et al. (2012), Bailey (2006), and Scott & Irish (2017).

Tooth Type	Trait	Definition	Grades	Description
UC	**Tuberculum dentale**	Presence of tubercles, ridges, or cusp-like features expressed in the cingular region of the lingual surface.	0	No expression, resulting in a smooth surface.
			1	Faint ridging
			2	Trace ridging
			3	Strong ridging
			4	Pronounced ridging
			5	Weakly developed cuspule with a free apex.
			6	Strong cusp with a free apex.
	**Mesial ridge**	Relative development of the mesial marginal ridge on the lingual surface compared to the distal ridge.	0	Mesial and distal ridges are the same size and not attached to the tuberculum dentale.
			1	Mesial ridge is larger than the distal and it may be weakly attached to the tuberculum dentale.
			2	Mesial ridge is larger than the distal and is moderately attached to the tuberculum dentale.
			3	Mesial ridge is much larger and incorporates the tuberculum dentale. (Morris' type.)
	**Distal accessory ridge**	Ridge development in the distolingual fossa, between the tooth apex and the distolingual marginal ridge.	0	The ridge is absent.
			1	The ridge shows a faint expression.
			2	The ridge shows a slight expression.
			3	The ridge shows moderate development.
			4	The ridge is strongly developed.
			5	The ridge is pronounced.
	**Shovel shape**	The presence of lingual marginal ridges.	0	Marginal ridges are not expressed.
			1	Faint shovel shape
			2	Trace of shovel shape. Elevations of the marginal ridges are easily seen.
			3	Moderate shovel shape
			4	Pronounced shovel shape
			5	Strong shovel shape
UP3, UP4	**Essential crest**	Presence of the essential crest on the buccal or lingual cusp. Degree of expression and location (buccal/lingual) are scored	0	The crest is absent.
			1	The crest is present.
			2	The crest is bifurcated.
	**Accessory ridge (MaxPAR)**	The presence of accessory ridges on the buccal cusp. Degree of expression and location (mesial/distal) are scored.	0	The ridge is absent.
			1	The ridge is present.
	**Transverse crest**	Expression of a transverse crest connecting the main cusps of the premolar.	0	The crest is absent.
			1	The crest is weak or it is interrupted by the sagittal fissure.
			2	The crest is pronounced or the sagittal fissure is erased.
	**Accessory cusps**	The presence of an accessory marginal tubercle in form of a bulge or free-standing accessory tubercle on the marginal ridge. Location (mesial/distal) is scored.	0	The cusp is absent.
			1	The cusp is present.

SOM Table S2: Sample used in the comparison of non-metric features of upper permanent canines (UC), third premolars (UP3), and fourth premolars (UP4).

Groups		Sites	Source
Homo sapiens	early	Jebel Irhoud, Misliya, Qafzeh, Skhul, Sea Harvest, Hoedjies Punt, Klasies River Mouth, Equus Cave, Die Kelders	a-c, e
	later	Upper Paleolithic individuals from Les Abeilles, Abri Blanchard, Abri Labattut, Abri Pataud, Arcy-sur-Cure, Brno, Dolní Věstonice, Farincourt, Fourneau du Diable, Fontechevade, Grotte des Rois, Gough's Cave, Isturitz, La Chaud, La Ferrassie, La Gravette, La Madeleine, Laugerie-Basse, La Vachons, Miesslingtal, Mladeč, Peștera cu Oase, Oberkassel, Pavlov, Peche de la Boissiere, Roc-de-Combe, Solutré, St. Germain-la-Rivière; Epipaleolithic individuals from North West Africa; Mesolithic and Neolithic individuals from France recent individuals from globally distributed populations	a, d-e
Homo neanderthalensis		Arcy-sur-Cure (Grotte du Renne, Grotte de l'Hyène, Grotte du Loup, Grotte du Bison), La Quina, Malarnaud, Petit-Puymoyen, Pinilla del Valle, Cabezo Gordo, Engis, Fondo Cattíe, Saccopastore, Tabun, Krapina, Le Moustier, Monsempron, Saint-Césaire, Shanidar, Sidrón, Hortus, Grotta Breuil, Monte Fenera, Guattari, Devil's Tower Gibraltar, Regourdou, Roc de Marsal, Taddeo, Melpignano, Combe-Grenal, Vindija, Kůlna, Ochoz, Kebara, Amud, Ehringsdorf, Spy, Shanidar	d-e
**Middle Pleistocene**	Europe	Arago, Mauer, Mountmarin, Pontnewydd, Steinheim, Petralona, Fonta Ranuccio, Atapuerca-SH	d-e

a: Bailey, 2006; b: Hublin et al., 2017; c: Hershkovitz et al., 2018; d: Martinón-Torres et al., 2012; e: Bailey, 2002

SOM Table S3: Detailed list of samples underlying the comparison of dental dimensions summarized in SOM Tables S7 and visualized in Figure 3 and SOM Figure S4.

Groups		Sites	Source
Homo sapiens	Aterian	(Grotte des) Contrebandiers T5, Dar-es-Soltane II H6	a
	early	Jebel Irhoud, Misliya, Qafzeh, Skhul	i-m
	later	Upper Paleolithic individuals from Bacho Kiro, Vindija (Aurignacian), Dolní Věstonice, Mladeč, Pavlov, Predmostí, Abri Pataud, Cap Blanc, Le Peyrat, Grotte de la Balauziere, St. Germain-la-Rivière, Arene Candide, Barma Grande, Grotte des Enfants, Grotta Paglicci, Cisterna, Kent's Cavern, La Rochette, Cro-Magnon, Les Rois, Le Placard;<br>Epipaleolithic individuals from Taforalt, Morocco, and Afalou, Algeria;<br>recent individuals from Australia, India, Europe, Africa, Asia	b-h, n
Homo neanderthalensis		Scladina, Grotte du Bison, La Chaise-de-Vouthon – Abri Bourgeois-Delaunay & Abri Suard, La Quina, Marillac, Monsempron, Moula-Guercy, Shanidar, Amud, Bolomor cave, Palomas 1, Obi-Rakhmat, Le Moustier, Saccopastore, Spy, Tabun, Kůlna, Combe-Grenal, Kalmakia, La Fate, El Salt, Ciota Ciara cave (Monte Fenera), Grotte Boccard, La Ferrassie, Feldhofer Grotte, Taddeo cave, Krapina	b,m,o-aj
**Middle Pleistocene**	Europe	Atapuerca-SH, Arago, Petralona, Steinheim, Montmaurin, Pontnewydd	m, ak-ap
	NW Africa	Rabat (Kébibat), Thomas Quarries, Tighennif (Ternifine; isolated)	am, ao

a: Hublin et al., 2012; b: Voisin et al., 2012; c: Frayer, 1978; d: Vallois & Billy 1965; e: Patte, 1968; f: Blanchard et al 1974; g: Trinkaus et al 2011; h: Violette et al 2015; i: Hublin et al., 2017; j: Hershkovitz et al., 2018; k: Vandermeersch, 1981; l: Tillier, 2014; m: Wolpoff, 1971; n: personal communication with S. E. Bailey; o: Wolpoff, 1979; p: Benazzi et al., 2011; q: Tillier et al., 2013; r: Condemi, 2001; s: Verna, 2006; t: Genet-Varcin, 1976; u: Becam et al., 2019; v: Garralda et al., 2020; w: Coulonges et al., 1952; x: Smith et al., 2006; y: Hlusko et al., 2013; z: Harvati et al., 2013; aa: Trinkaus, 1983; ab: Sakura, 1970; ac: Arsuaga et al., 2012; ad: Pinalla & Trinkaus, 2017; ae: Glantz et al., 2008; af: Jelinek 2012; ag: Villa & Giacobini, 1996; ah: Garralda et al., 2014; ai: Maureille et al., 2008; aj: Toussaint, 2014; ak: Martinón-Torres et al., 2012; al: Bermúdez de Castro et al., 2019; am: Bermúdez de Castro, 1986; an: Compton & Stringer, 2015; ao: Raynal et al., 2010;

SOM Table S4: Sample used in the enamel-dentine junction (EDJ) analyses of upper permanent canines (UC), third premolars (UP3), and fourth premolars (UP4).

Group		Geological Age	Country of Origin	UC	UP3	UP4	Individuals[†]	Abbreviation for Fossil Individuals	Repository/ housing institution of CT scans
Mugharet el'Aliya		Late Pleistocene	Morocco	1	1	1	El'Aliya	El'Aliya	Peabody Museum, Harvard
Homo sapiens	later	Holocene	Tunisia	6	6	9			Osteological Collection University of Tübingen (H. Rathmann)
			Egypt	2	4	8			
			Tanzania	1	4	3			
			Germany		9	7			
		Late Pleistocene	France		1	1	La Rochette	LR	
						1	Abri Pataud	AP	Muséum National d'Histoire Naturelle (MNHN; A. Balzeau, D. Grimaud-Hervé)
	Aterian		Morocco		1	1	Dar-es-Soltane II H6	DS6	Max Planck Institut für evolutionäre Anthropologie (MPI EVA; J.-J. Hublin, P. Gunz)
						1	(Grotte des) Contrabandiers T5	T5	
	early		Israel		1		Qafzeh 10	QA10	ESRF heritage database (Smith et al., 2010)
				1	1	1	Qafzeh 15	QA15	
		Middle Pleistocene	Morocco	1			Jebel Irhoud 21	IR21	Institut National des Sciences de l'Archéologie et du Patrimoine (INSAP; A. Ben-Ncer) & Max Planck Institut für evolutionäre Anthropologie (MPI EVA; J.-J. Hublin, P. Gunz)
Homo neanderthalensis		Late Pleistocene	France			1	Abri Bourgeois-Delaunay 14	BD14	NESPOS database[‡] (R. Macchiarelli)
				1	1	1	La Quina H18	LQ18	ESRF heritage database (Smith et al., 2010; C. Verna, C. Schwab)
				1	1	1	Le Moustier 1	LM	Museum für Naturkunde, Museum für Vor- und Frühgeschichte, Staatliche Museen zu Berlin (E. Dutkiewicz, K. Mahlow)
		Middle Pleistocene	Croatia	5			Krapina 76, 102, 103, 141, 191	Kr76, Kr102, Kr103, Kr141, Kr191	NESPOS database[‡]
					5		Krapina 39, 45, 53, 112, 116	Kr39, Kr45, Kr53, Kr112, Kr116	
						8	Krapina 41, 42, 44, 46, 47, 49, 115, 117	Kr41, Kr42. Kr44. Kr46, Kr47, Kr49, Kr115, Kr117	

[†]for Holocene non-fossil individuals only the number of used individuals is provided in columns UC, UP3, and UP4.

[‡]NESPOS database no longer available; Krapina scans now available through NM digital archive

SOM Table S5: Landmark definitions for enamel-dentine junction (EDJ) analyses of upper permanent canines (UC), third premolars (UP3), and fourth premolars (UP4).

Dataset	LM Number in Figures	Landmark Definition
UP3, UP4	1	Mesial fovea or point where the central groove intersects the mesial foveal grooves on the enamel*
	2	Distal fovea or point where the central groove intersects the distal foveal grooves on the enamel†
	3	Deepest point on mesial fovea on the EDJ
	4	Deepest point on distal fovea on the EDJ
	5	Tip of the buccal dentine horn (paracone) on the EDJ
	6	Tip of the lingual dentine horn (protocone) on the EDJ
UC	7	Tip of the dentine horn on the EDJ
	8	Most cervical point of the lingual surface at the intersection between the mesial and distal curves on the EDJ. In the case of a present tuberculum dentale, located distally to the tuberculum

†following the definitions provided in Gómez-Robles et al. 2011

SOM Table S6: Detailed list of the comparative sample underlying the perikymata analysis of the upper permanent canine (UC). R = right. L = left. NA = undetermined.

**Middle Pleistocene Europe**†		***Homo neanderthalensis***		**later *Homo sapiens***	
Catalog number	Side	Individuals/Sites	Side	Individuals/Sites	Side
44	L	Genay 1	R	Abri Pataud 22	L
94	NA	Genay 1	L	Aurensan di	R
144	R	Hortus II-III	R	Grotte de Bedeihac	R
163	L	Hortus II-III	L	Estagel 1	R
558	R	Hortus VIII	L	Estagel 1	L
818	L	Hortus VIII	R	Laugerie-Basse	R
825	L	Hortus IX	R	Le Placard	L
955	L	Krapina 36	R	Mas d'Azil	L
958	L	Krapina 37	L	Saulges Mx 1	L
1475	R	Krapina 56	R	Solutré Mx 2	R
1757	L	Krapina 76	R	Solutré Mx 2	L
1758	R	Krapina 103	L	Soultré di	R
1942	L	Krapina 139	L	St. Germain-la-Rivière 10	R
2151	L	Krapina 141	R	St. Germain-la-Rivière 5	R
2207	R	Krapina 142	L	Tarté 1	L
2388	R	Krapina 144	L		
2392	L	Krapina 146	L		
3191	R	Krapina 147	L		
		Krapina Mx 45.1	R		
		Krapina Mx E	L		
		Krapina Mx F	R		
		La Ferrassie II	R		
		La Quina 5	R		
		La Quina 5	L		
		La Quina 17	R		
		Monsempron III	R		
		Saccopastore II	R		
		Saccopastore II	L		
		Vindija 12.5	R		

†All teeth from the European Middle Pleistocene are from the site of Sima de los Huesos, Atapuerca, Spain.

SOM Table S7: Comparisons of dental crown dimensions for upper permanent canines (UC), third premolars (UP3), and fourth premolars (UP4). MD = mesiodistal diameter. BL = buccolingual diameter. Measurements in mm and mm^2, respectively, and values rounded to two decimals. The sample compositions are listed in detail in SOM Table S3.

				Homo sapiens				Middle Pleistocene	
			**El'Aliya**	Aterian	early	later	***Homo neanderthalensis***	Europe	NW Africa
UC	MD	N			13	143	40	26	3
		x̄	8.20		8.60	7.82	8.63	8.73	9.13
		σ			0.69	0.59	0.68	0.40	0.40
	BL	N			13	143	40	26	3
		x̄	10.90		9.24	8.69	9.88	9.70	9.93
		σ			0.68	0.66	0.67	0.54	0.12
	MD*BL	N			13	143	40	26	3
		x̄	89.38		79.58	68.28	85.47	84.71	90.72
		σ			9.98	10.69	11.38	7.03	4.03
UP3	MD	N		1	9	192	38	23	4
		x̄	7.70	8.40	7.77	7.1	7.83	7.89	8.48
		σ			0.65	0.60	0.68	0.57	0.21
	BL	N		1	9	192	38	23	4
		x̄	10.30	10.00	10.49	9.48	10.75	10.53	11.65
		σ			0.65	0.82	0.64	0.70	0.44
	MD*BL	N		1	9	192	38	23	4
		x̄	79.31	84.00	81.73	67.73	84.51	83.38	98.79
		σ			10.98	10.82	11.78	11.31	5.81
UP4	MD	N		2	9	207	37	23	2
		x̄	8.00	8.95	7.18	6.86	7.34	7.65	8.15
		σ		0.35	0.56	0.71	0.84	0.59	0.21
	BL	N		2	9	207	37	23	2
		x̄	11.10	11.50	10.19	9.61	10.35	10.33	11.10
		σ		0.50	0.98	0.81	0.64	0.77	0.14
	MD*BL	N		2	9	207	37	23	2
		x̄	88.80	102.75	73.39	66.32	76.33	79.45	90.48
		σ		0.45	10.81	12.08	12.86	11.77	3.51

SOM Table S8: Analyses of variance (ANOVA) of the EDJ datasets for upper canines (UC), upper third (UP3) and fourth premolars (UP4). Sample compositions are provided in SOM Table S4. All values rounded to three decimals. Significant correlations highlighted as bold p-values.

Tooth type	Variable	DoF	R^2	F-value	Z-value	p-value
**UC**	log (CS)	1	0.172	3.629	2.611	**0.033**
	group	3	0.165	1.169	0.574	0.293
**UP3**	log (CS)	1	0.072	2.825	2.034	**0.021**
	group	3	0.162	2.121	2.280	**0.011**
**UP4**	log (CS)	1	0.120	6.187	4.214	**<0.001**
	group	3	0.124	2.126	2.582	**0.007**

SOM Table S9: Perikymata counts by decile for the upper permanent canine (UC). The sample compositions are listed in detail in SOM Table S6. Measurements values rounded to one decimal.

		**El'Aliya**	**Middle Pleistocene Europe**	***Homo neanderthalensis***	**later *Homo sapiens***
**Decile 5**	N		9	17	10
	$\bar{x}$	15.0	14.3	12.6	15.2
	σ		2.5	2.3	2.3
	range		11-17	9-17	11-19
**Decile 6**	N		14	21	10
	$\bar{x}$	17.0	15.5	14.0	17.3
	σ		3.5	2.7	2.3
	range		10-21	9-20	14-21
**Decile 7**	N		17	26	14
	$\bar{x}$	20.0	17.9	15.3	19.2
	σ		3.5	3.0	2.2
	range		12-23	11-23	16-23
**Decile 8**	N		16	28	15
	$\bar{x}$	22.0	20.6	17.1	26.5
	σ		3.6	2.4	4.6
	range		14-26	14-24	20-35

References

Arsuaga, J.-L., Fernández Peris J., Gracia-Téllez A., Quam R., Carretero J.M., Barciela González V., Blasco R., Cuartero F., & Sañudo P. (2012). Fossil human remains from Bolomor Cave (Valencia, Spain). *Journal of Human Evolution*, 62 (5), 629-639.

Bailey, S. E. (2002). Neandertal dental morphology: implications for modern human origins. Ph.D. dissertation, Arizona State University.

Bailey, S. E. (2006). Beyond shovel-shaped incisors: Neandertal dental morphology in a comparative context. *Periodicum Biologorum* 108 (3), 253-267. https://hrcak.srce.hr/83097.

Becam, G., Verna C., Gómez-Robles A., Gómez-Olivencia A., Albessard L., Arnaud J., Frelat M. A., Madelaine S., Schwab C., Souday C., Turq A., & Balzeau A. (2019). Isolated teeth from La Ferrassie: Reassessment of the old collections, new remains, and their implications. *American Journal of Physical Anthropology*, 169 (1), 132-142.

Benazzi, S., Viola, B., Kullmer, O., Fiorenza, L., Harvati, K., Paul, T., Gruppioni, G., Weber, G.W., & Mallegni, F. (2011). A reassessment of the Neanderthal teeth from Taddeo cave (southern Italy). *Journal of Human Evolution*, 61 (4), 377-387.

Bermúdez de Castro, J. M. (1986). Dental Remains from Atapuerca (Spain) I. Metrics. *Journal of Human Evolution*, 15, 265-287.

Bermúdez de Castro, J. M., Martinón-Torres, M., de Pinillos, M. M., García-Campos, C., Modesto-Mata, M., Martín-Frances, L., & Arsuaga, J. L. (2019). Metric and morphological comparison between Arago (France) and Atapuerca-Sima de los Huesos (Spain) dental samples, and the origin of Neanderthals. *Quaternary Science Reviews*, 217, 45-61. Doi: 10.1016/j.quascirev.2018.04.003.

Bernal, V., Perez, S. I., Gonzalez, P. N., & Diniz-Filho, J. A. F. (2010). Ecological and evolutionary factors in dental morphological diversification among modern human populations from southern South America. *Proceedings of the Royal Society B*, 277, 1107-1112. Doi: 10.1098/rspb.2009.1823.

Blanchard R., Peyrony D., & Vallois H. V. (1974). Le gisement et squelette de Saint-Germain- la-Rivière. *Archives de l'Institut de Paléontologie Humaine*, 34, 1-112.

Compton, T., & Stringer, C. (2015). The morphological affinities of the Middle Pleistocene hominin teeth from Pontnewydd Cave, Wales. *Journal of Quaternary Science*, 307 (7), 713-730. Doi: 10.1002/jqs.2811.

Condemi, S. (2001). Les néandertaliens de La Chaise. *Edition du CTHS*, Paris, 15, 178.

Coulonges, L., Lansac A., Piveteau J., & Vallois H. V. (1952). Le gisement préhistorique de Monsempron (Lot-et-Garonne). *Annales de Paléontologie*, 38, 83-120.

Frayer, D. W. (1978). *Evolution of the Dentition in Upper Paleolithic and Mesolithic Europe*. Lawrence, Kansas: University of Kansas, Publications in Anthropology n°10.

Garralda, M. D., Galván, B., Hernández, C. M., Mallol, C., Gómez, J. A., & Maureille, B. (2014). Neanderthals from El Salt (Alcoy, Spain) in the context of the latest Middle Palaeolithic populations from the southeast of the Iberian Peninsula. *Journal of Human Evolution*, 75, 1-15.

Garralda, M.D., Maureille B., Le Cabec A., Oxilia G., Benazzi S., Skinner M.M., Hublin J.-J., & Vandermeersch B. (2020). The Neanderthal teeth from Marillac (Charente, Southwestern France): morphology, comparisons and paleobiology. *Journal of Human Evolution*, 138, 102683.

Genet-Varcin, E. (1976). Étude de dents humaines isolées provenant de La Chaise de Vouthon (Charente) (Fin). *Bulletins et Mémoires de la Société d'anthropologie de Paris*, 13 (3), 243-259.

Glantz, M., Viola B., Wrinn P., Chikisheva T., Derevianko A., Krivoshapkin A., Islamov U., Suleimanov R., & Ritzman T. (2008). New hominin remains from Uzbekistan. *Journal of Human Evolution*, 55 (2), 223-237.

Gómez-Robles, A., Martinón-Torres, M., Bermudez de Castro, J. M., Prado-Simón, L., & Arsuaga, J. L. (2011). A geometric morphometric analysis of hominin upper premolars. Shape variation and morphological integration. *Journal of Human Evolution*, 61 (6), 688-702.

Harvati, K., Darlas A., Bailey S. E., Rein T. R., El Zaatari S., Fiorenza L., Kullmer O., & Psathi E. (2013). New Neanderthal remains from Mani peninsula, Southern Greece: The Kalamakia Middle Paleolithic cave site. *Journal of Human Evolution*, 64 (6), 486-499.

Hershkovitz, I., Weber, G. W., Quam, R., Duval, M., Grün, R., Kinsley, L., Ayalon, A., Bar-Matthews, M., Valladas, H., Mercier, N., Arsuaga, J. L., Martinón-Torres, M., Bermúdez de Castro, J. M., Fornai, C., Martín-Francés, L., Sarig, R., May, H., Krenn, V. A., Slon, V., Rodríguez, L., García, R., Lorenzo, C., Carretero, J. M., Frumkin, A., Shahack-Gross, R., Bar-Yosef Mayer, D. E., Cui, Y., Wu, X., Peled, N., Groman-Yaroslavski, I., Weissbrod, L., Yeshurun, R., Tsatskin, A., Zaidner, Y., & Weinstein-Evron, M. (2018). The earliest modern humans outside Africa. *Science*, 359, 456-459. Doi: 10.1126/science.aap8369.

Hlusko, L. J., Carlson J. P., Guatelli-Steinberg D., Krueger K. L., Mersey B., Ungar P. S., & Defleur A. (2013). Neanderthal Teeth from Moula-Guercy, Ardèche, France. *American Journal of Physical Anthropology*, 151 (3), 477–491

Hublin, J.-J., Verna, C., Bailey, S. E., Smith, T., Olejniczak, A., Sbihi-Alaoui, F. Z., & Zouak, M. (2012). Dental Evidence from the Aterian Human Populations of Morocco, In: Hublin, J.-J., McPherron, S. P. (Eds.), *Modern Origins: A North African Perspective*. Springer, Berlin, pp. 189-204. Doi: 10.1007/978-94-007-2929-2_13.

Hublin, J.-J., Ben-Ncer, A., Bailey, S. E., Freidline, S. E., Neubauer, S., Skinner, M. M., Bergmann, I., le Cabec, A., Benazzi, S., Harvati, K., & Gunz, P. (2017). New fossils from Jebel Irhoud, Morocco, and the pan-African origin of *Homo sapiens*. *Nature*, 546, 289–292. Doi: 10.1038/nature22336.

Jenlínek, J. (2012). The discovery of a Neanderthal jawbone (Kůlna I) in Kůlna Cave, Morovia. *Athropologie (1962-)*, 50 (2), 147-166.

Martinón-Torres, M., Bermúdez de Castro, J. M., Gómez-Robles, A., Prado-Simón, L., & Arsuaga, J. L. (2012). Morphological description and comparison of the dental remains from Atapuerca-Sima de los Huesos site (Spain). *Journal of Human Evolution*, 62, 7-58. Doi: 10.1016/j.jhevol.2011.08.007.

Maureille, B., Djindjian F., Garralda M. D., Mann A., & Vandermeersch B. (2008). Les dents moustériennes de la Grotte Boccard, Lieu-dit Bas-De-Morant (Commune de Créancey, Côte-d’Or, Bourgogne). *Bulletins et Mémoires de la Société d’Anthropologie de Paris*, 20 (1-2), 59-78.

Patte, E. (1968). L’homme et la femme de l’Azilien de Saint-Rabier (fouilles Cheynier). *Mémoires du Muséum national d’histoire naturelle série C*, XIX, 1–56.

Pinilla, B., & Trinkaus E. (2017). The Palomas dental remains - size and proportions. In: Trinkaus E., Walker M.J. (Eds.), *The people of Palomas - Neandertals from the Sima de las Palomas del Cabezo Gordo, Southeastern Spain*. College Station, Texas: Texas A&M University Press, pp. 89-104.

Raynal, J.-P., Sbihi-Alaoui, F.-Z., Mohib, A., el Granoui, M., Lefèvre, D., Texier, J.-P., Geraads, D., Hublin, J.-J., Smith, T., Tafforeau, P., Zouak, M., Grün, R., Rhodes, E. J., Eggins, S., Daujeard, C., Fernandes, P., Gallotti, R., Hossini, S., & Queffelec, A. (2010). Hominid Cave at Thomas Quarry I (Casablanca, Morocco): recent findings and their context. *Quaternary International*, 223-224, 369-382. Doi: 10.1016/j.quaint.2010.03.011.

Richter, D., Grün, R., Joannes-Boyau, R., Steele, T. E., Amani, F., Rué, M., Fernandes, P., Raynal, J.-P., Geraads, D., Ben-Ncer, A., Hublin, J.-J., & McPharron, S. (2017). The age of the hominin fossils from Jebel Irhoud, Morocco, and the origins of the Middle Stone Age. *Nature*, 546, 293-296. Doi: 10.1038/nature22335.

Sakura, H. (1970). Dentition of the Amud man. In: Suzuki, H., Takai, F. (Eds.), *The Amud Man and his Cave Site*. The University of Tokyo, Tokyo, pp. 207-229

Scott, G. R., & Irish, J. D. (2017). *Human Tooth Crown and Root Morphology: The Arizona State University Dental Anthropology System*. Cambridge University Press, Cambridge.

Smith, F. H., Smith, M. O., & Schmitz, R.W. (2006). Human skeletal remains from the 1997 and 2000 excavations of the cave deposits derived from Kleine Feldhofer Grotte in the Neander Valley, Germany. In: Schmitz, R.W. (Eds.), *Neanderthal 1856-2006*. Mainz am Rhein: Verlag Philipp von Zabern, Reinische Ausgrabungen Band 58, pp. 187-246.

Smith, T. M., Tafforeau, P., Reid, D. J., Grün, R., Eggins, S., Boutakiout, M., & Hublin, J.-J. (2007). Earliest evidence of modern human life history in North African early *Homo sapiens*. *Proceedings of the National Academy of Sciences*, 104 (15), 6128-6133. Doi: 10.1073pnas.0700747104.

Smith, T.M., Tafforeau, P., Reid, D.J., Pouech, J., Lazzari, V., Zermeno, J.P., Guatelli-Steinberg, D., Olejniczak, A.J., Hoffman, A., Radovčić, J., Makaremi, M. (2010). Dental evidence for ontogenetic differences between modern humans and Neanderthals. *Proceedings of the National Academy of Sciences*, 107 (49), 20923-20928.

Tillier, A. M., Sansilbano-Collilieux M., David F., Enloe J. G., Girard M., Hardy M., D'iatchenko V., Roblin-Jouve A., & Tolmie C. (2013). Les vestiges néanderthaliens provenant des niveaux moustériens I et J de la Grotte du Bison à Arcy-sur-Cure (Yonne): bilan actuel. *Bulletins et Mémoires de la Société d'Anthropologie de Paris*, 25 (1-2), 39-54.

Tillier, A.-M. (2014). New Middle Paleolithic Hominin Dental Remains from Qafzeh, Israel. *Paléorient* 40 (1), 13-24.

Toussaint, M. (2014). The dentition of the Scladina 1-4A juvenile Neandertal. In: Toussaint, M., Bonjean, D. (Eds.), *The Scladina I-4A Juvenile Neandertal (Andenne, Belgium) Palaeoanthropology and Context*. Études et Recherches Archéologiques de l'Université de Liège, 134, pp.233-306.

Trinkaus, E. (1983). *The Shanidar Neandertals*. New York: Academic Press.

Trinkaus, E., Bailey, S. E., Davis, S. J. M., & Zilhao, J. (2011). The Magdalenian Human Remains from the Galeria da Cisterna (Almonda karstic systen, Torres Novas, Portugal) and their Archeological Context. *O Archueólo* Português, V (1), 395-413.

Vallois, H. V., & Billy, G. (1965). Nouvelles recherches sur les hommes fossiles de l'abri de Cro-Magnon. *L'Anthropologie*, 69, 47-74.

Vandermeersch, B. (1981). *Les hommes fossiles de Qafzeh, Israel*. Paris: CNRS.

Verna, C. (2006). Les restes humains moustériens de la station Amont de la Quina – (Charente, France). Contexte archéologique et constitution de l'assemblage. Étude morphologique et métrique des restes crânio-faciaux. Apport à l'étude de la variation néandertalienne. Ph.D. dissertation: Université de Bordeaux 1.

Villa, G., & Giacobini G. (1996). Neandertal teeth from alpine caves of Monte Fenera (Piedmont, northern Italy): description of the remains and microwear analysis. *Anthropologie (Brno)*, 34 (1/2), 55-67.

Villotte, S., Chiotti, L., Nespoulet, R., & Henry-Gambier, D. (2015). Étude anthropologique des vestiges humains récemment découverts issus de la couche 2 de l'abri Pataud (Les Eyzies-de-Tayac-Sireuil, Dordogne, France). *Bulletins et Mémoires de la Société d'Anthropologie de Paris* 27, 158-188. Doi: 10.1007/s13219-015-0128-3.

Voisin, J.-L., Condemi, S., Wolpoff, M. H., & Frayer, D. W. (2012). A new online database (https://anthropologicaldata.free.fr) and a short reflection about the productive use of compiling internet data. *PaleoAnthropology*, 2012, 241-244. Doi: 10.4207/PA.2012.ART76.

Wolpoff, M. H. (1971). *Metric Trends in Hominid Dental Evolution*. Case Western University Press: Cleveland.

Wolpoff, M. H. (1979). The Krapina dental remains. *American Journal of Physical Anthropology*, 50 (1), 67-113
